# Beyond CAR-T Cells: exploring CAR-NK, CAR-M, and CAR-γδ T strategies in solid tumor immunotherapy

**DOI:** 10.3389/fimmu.2025.1675807

**Published:** 2025-10-16

**Authors:** Yingjie Hou, Shihua Hu, Chong Liu, Xin Chen, Yuru Wang, Youzherui Li, Zihe Fu, Chunjing Feng, Yanhua Gong, Zichuan Liu, Shouchun Peng

**Affiliations:** ^1^ School of Disaster and Emergency Medicine, Tianjin University, Tianjin, China; ^2^ Tianjin University and Health-Biotech United Group Joint Laboratory of Innovative Drug Development and Translational Medicine, School of Pharmaceutical Science and Technology, Faculty of Medicine, Tianjin University, Tianjin, China; ^3^ Thinking Biomed (Beijing) Co., Ltd, Beijing Economic and Technological Development Zone, Beijing, China; ^4^ Department of Respiratory Medicine, Jinnan Hospital, Tianjin University, Tianjin, China; ^5^ Jiangxi Engineering Research Center for Stem Cell, Jiangxi Health-Biotech Stem Cell Technology Co., Ltd., Shangrao, Jiangxi, China; ^6^ State Key Laboratory of Synthetic Biology, Tianjin University, Tianjin, China; ^7^ Department of Respiratory Medicine, Tianjin Jinnan Hospital, Tianjin, China

**Keywords:** chimeric antigen receptor, macrophage cells, NK cells, solid tumor, γδT cells

## Abstract

Adoptive cell therapy (ACT) employing chimeric antigen receptor (CAR) engineering represents a transformative advancement in cancer immunotherapy. CAR-T cell therapies have demonstrated significant clinical success in hematological malignancies, yet their application to solid tumors faces persistent challenges. Key limitations include the paucity of tumor-specific antigens, poor intratumoral infiltration, immunosuppressive tumor microenvironment (TME), and treatment-related toxicities such as cytokine release syndrome (CRS) and neurotoxicity. In contrast, CAR natural killer (CAR-NK) cells show promise in solid tumors such as ovarian, pancreatic, and glioblastoma, with encouraging preclinical and early clinical evidence, although limited persistence and antigen heterogeneity remain major challenges. Unlike CAR-T cells, CAR-NK therapies mediate tumor clearance through both cytotoxic (e.g., granzyme/perforin release) and cytokine-mediated mechanisms while mitigating toxicity risks. Their lack of human leukocyte antigen (HLA) dependency enables “off-the-shelf” manufacturing from allogeneic donors, circumventing patient-specific production bottlenecks. CAR-macrophage (CAR-M) therapies further address solid tumor barriers by leveraging innate phagocytic clearance, antigen-presenting functions, and TME penetration. Macrophages inherently infiltrate hypoxic tumor regions and remodel stromal barriers, enabling CAR-Ms to synergize with adaptive immunity by cross-priming T cells. Preclinical models highlight CAR-M efficacy in depleting immunosuppressive tumor-associated macrophages (TAMs) and reversing TME-driven immune evasion. Emerging CAR- Gamma-Delta T (CAR-γδ T) cell therapies combine CAR-mediated antigen specificity with the intrinsic tumoricidal activity of γδ T cells, which recognize stress-induced ligands independently of major histocompatibility complex (MHC) presentation. This dual-targeting capability enhances tumor selectivity while reducing on-target/off-tumor toxicity. This review systematically examines cellular sources, mechanistic advantages and clinical progress. By evaluating these platforms’ complementary strengths, we propose rational strategies for integrating CAR-NK, CAR-M, and CAR-γδ T cells into tailored therapeutic regimens for solid tumors.

## 1 Introduction

Cancer stands as a prominent cause of mortality globally. Traditional modalities such as surgery, radiation therapy, and chemotherapy constitute the cornerstone of treating early to mid-stage cancers (1). However, the substantial adverse effects associated with chemotherapy and radiation have led to the emergence of immunotherapy as a promising alternative for various cancer types. This approach leverages the immune system’s capacity to zero in on cancer cells (2). Tumor cells evade immune surveillance through various mechanisms, leading to uncontrolled proliferation driven by factors such as genetic mutations, chemical carcinogens, physical damage, and viral infections. A major challenge in cancer therapy is minimizing the collateral damage to healthy tissues, which often occurs during treatments such as chemotherapy and radiation therapy. Immune cells play a critical role in cancer defense, with T cells recognizing abnormal cells by detecting antigen peptides presented by major histocompatibility complex I (MHC-I). However, tumor cells can evade immune surveillance by interfering with the immune recognition process, such as downregulating MHC-I expression or altering antigen presentation, which contributes to their uncontrolled proliferation and metastasis (3). Antibody-antigen interactions are fundamental to immune recognition in humans. By grafting antibodies that bind to tumor cell surface receptors onto cell membranes, “smart cells” known as CAR-engineered cells can be created. These cells efficiently recognize and eliminate cancer cells. Introducing these modified cells into patients for cancer treatment is called adoptive cell therapy (4). Engineered immune cells, particularly CAR-T constructs, have emerged as an innovative strategy for tumor eradication. The conventional manufacturing protocol for CAR-T therapies commences with leukapheresis, a procedure to isolate peripheral blood mononuclear cells (PBMCs) from the patient. Following isolation, sequential processing steps are performed: cell washing to remove plasma contaminants, T-cell activation via CD3/CD28 agonists, viral/non-viral gene transfer for CAR integration, ex vivo expansion in cytokine-enriched media, and formulation into a final cryopreserved product for single-dose infusion (5–8). This labor-intensive process spans 10–20 days and necessitates multiple transfers across sterile barriers, introducing logistical complexities. Two critical limitations undermine the druggability of autologous CAR-T therapies. First, patient-specific production contradicts the pharmaceutical industry’s emphasis on standardized production protocols, complicating batch consistency and regulatory quality control. Second, while allogeneic “off-the-shelf” CAR-T strategies aim to circumvent these issues, clinical trials reveal persistent challenges, including immune rejection, limited persistence, and suboptimal efficacy in heterogeneous tumor microenvironments (9, 10). These hurdles highlight the need for innovative manufacturing frameworks to harmonize personalized efficacy with scalable production. A second critical limitation lies in the heterogeneous variable expansion and persistence of CAR-T therapies, which complicate their clinical predictability and safety. Following single-dose administration, CAR-T cells exhibit variable expansion kinetics and tissue distribution, a phenomenon termed “post-administration pharmacokinetic variability.” This unpredictability arises from patient-specific factors (e.g., T-cell fitness, tumor burden) and intrinsic CAR-T behaviors, such as activation-induced exhaustion or uncontrolled clonal proliferation (11, 12). These dynamics may lead to suboptimal therapeutic outcomes, including incomplete tumor clearance or severe adverse events such as CRS and immune effector cell-associated neurotoxicity syndrome (ICANS) (13, 14). CAR-T cell therapy has witnessed remarkable advancements in treating hematologic malignancies, yet it encounters hurdles when addressing solid tumors. The primary challenges include the complexities involved in manufacturing CAR-T cells, the scarcity of specific tumor antigens, inadequate infiltration of CAR-T cells into tumors, immune suppression within the tumor microenvironment, toxicity associated with treatment, and antigen escape mechanisms (15). To address these limitations, alternative CAR-engineered immune cells have emerged. CAR-NK cells leverage innate tumor-homing capacity and MHC-independent cytotoxicity, while CAR-Ms exploit phagocytic clearance and TME remodeling. Preclinical studies demonstrate synergistic antitumor efficacy for both modalities in solid tumors, accompanied by more favorable toxicity profiles compared to CAR-T cells (16, 17). Emerging CAR-γδ T cell therapies combine CAR-mediated antigen specificity with the intrinsic tumoricidal activity of γδ T cells, which recognize stress-induced ligands independently of MHC presentation. This dual-targeting capability enhances tumor selectivity while reducing on-target/off-tumor toxicity.

CAR-NK cell therapy involves the genetic modification of NK cells to express synthetic receptors that recognize tumor-specific antigens. Unlike T cells, NK cells mediate cytotoxicity through MHC-independent mechanisms, enabling them to target malignant cells without prior antigen sensitization—a hallmark of innate immunity (18). This intrinsic capability positions CAR-NK cells as promising candidates for solid tumor immunotherapy, offering advantages such as reduced risk of CRS and compatibility with “off-the-shelf” manufacturing (19). Preclinical studies have validated CAR-NK efficacy across diverse solid malignancies, including breast cancer, ovarian cancer (OC), pancreatic ductal adenocarcinoma, colorectal carcinoma, glioblastoma multiforme, hepatocellular carcinoma (HCC), and head and neck squamous cell carcinoma (HNSCC) (20–23). For instance, CAR-NK cells targeting human epidermal growth factor receptor 2 (HER2) in OC models demonstrated robust tumor regression via dual mechanisms: direct cytolytic activity and interferon-γ (IFN-γ)-mediated TME remodeling (21). CAR-NK cells, engineered to secrete Neo-2/15 (an IL-2Rβγ agonist), demonstrate enhanced cytotoxicity and persistence in immunosuppressive TME. They exhibit significant efficacy in pancreatic ductal adenocarcinoma (PDAC) and ovarian cancer by sustaining mitochondrial fitness via c-Myc/NRF1 activation, leading to reduced exhaustion and superior tumor control (24). Similarly, anti- mucin 1 (MUC1) CAR-NK cells exhibited potent suppression of pancreatic cancer progression *in vivo*, correlating with enhanced infiltration and reduced stromal barrier density (22). CAR-NK therapies can circumvent HLA compatibility requirements due to their innate MHC-independent recognition mechanisms, which promotes their ‘off-the-shelf’ applicability (16). However, the long-term persistence and efficacy of allogeneic CAR-NK cells can be limited by host immune rejection, mediated through mechanisms such as the development of anti-CAR antibodies or engagement of host NK cell inhibitory receptors. Strategies to mitigate this, including HLA editing and the use of transient immunosuppressive conditioning regimens, are under active investigation to fully realize the ‘off-the-shelf’ potential of CAR-NK products.

Concurrently, CAR-M therapy has emerged as an innovative strategy leveraging the innate phagocytic and immunomodulatory functions of macrophages. These engineered cells are reprogrammed to express CARs, enabling targeted phagocytosis of malignant cells while remodeling the immunosuppressive TME via pro-inflammatory cytokine secretion (e.g., interleukin [IL]-12, tumor necrosis factor-α [TNF-α]) and antigen-presenting functions (25). Unlike CAR-T/NK cells, CAR-Ms uniquely degrade stromal barriers (e.g., fibrotic extracellular matrix) to enhance immune cell infiltration, synergizing with adaptive immunity to sustain antitumor responses (26). Robust preclinical evidence for CAR-M therapy against solid tumors has fueled interest attributed to the inherent abundance of macrophages within the TME and their critical involvement in facilitating tumor progression and metastatic dissemination (27, 28). Furthermore, CAR-M cells enhance phagocytic clearance of malignant cells and improve tumor antigen presentation to T lymphocytes (29). These dual mechanisms highlight their potential to serve as a transformative strategy in immuno-oncology by simultaneously targeting tumor cells and modulating immunosuppressive TME networks. Currently, CAR-M therapy shows promise for solid tumors but faces challenges: macrophage plasticity (M1/M2 polarization), short *in vivo* persistence, and difficulties in genetic modification. Including breast carcino CAR-NK therapies can circumvent HLA compatibility requirements due to their innate Mma, glioblastoma, and pancreatic adenocarcinoma (30). Early-phase clinical trials (e.g., NCT04660929) are underway to systematically evaluate the safety profile and antitumor activity of CAR-M therapies across multiple cancer types, with a focus on solid malignancies (ClinicalTrials.gov). Building upon the clinical success of CAR-T cell therapies and emerging progress in CAR-NK platforms, the scientific community has garnered significant interest in advancing CAR-M-based approaches for cancer immunotherapy. The development of CAR-M introduces novel therapeutic avenues for solid tumors through genetic engineering of human macrophages. By equipping these cells with tumor-targeting CAR constructs, researchers aim to amplify their innate phagocytic functions while optimizing their capacity for cross-presenting tumor-associated antigens (31).Despite encouraging preclinical/clinical progress, key challenges hinder CAR-NK, CAR-γδ T, and CAR-M efficacy in solid tumors: enhancing *in vivo* persistence within immunosuppressive microenvironments, improving tumor specificity to mitigate off-target toxicity, and optimizing functional potency. This review analyzes CAR design innovations, mechanistic distinctions and recent advancements to address these barriers. This review will first outline the fundamental barriers limiting CAR-T cell efficacy in solid tumors. It will then comparatively examine the unique mechanisms and advantages of three promising alternative platforms—CAR-NK, CAR-M, and CAR-γδ T cells—in overcoming these challenges, concluding with a perspective on their future clinical translation.

## 2 Immune cells

Innate immunity constitutes the primary frontline defense system, encompassing physical barriers, chemical mediators, and a diverse array of immune cells expressing pattern-recognition receptors (PRRs). Key cellular components include myeloid lineage cells—dendritic cells (DCs), macrophages, monocytes, neutrophils, eosinophils, basophils, and mast cells—as well as lymphoid subsets such as NK cells, NKT cells, γδ T cells, and mucosa-associated invariant T (MAIT) cells. These innate effectors restrict tumor progression through direct cytolytic activity against malignant cells or by orchestrating adaptive immunity via antigen presentation and cytokine signaling (32). In contrast to the innate system, which provides rapid but nonspecific responses, the adaptive immune system specifically targets and eradicates cancer cells by T and B cells (33). Antitumor immunotherapies, including adoptive cell transfer (34, 35), have undergone extensive validation and received clinical approval for diverse cancer types. Notably, CAR-T therapy has demonstrated remarkable efficacy against hematological malignancies, representing a major advancement in oncological treatment (36, 37). The CAR-T cell manufacturing process involves the genetic modification of autologous T lymphocytes to express engineered receptors (38). These CARs incorporate extracellular antigen-binding domains capable of tumor antigen recognition, coupled with intracellular signaling motifs that replicate T-cell receptor (TCR) activation pathways (39, 40). To date, six CAR-T therapies have received clinical approval for treating B-cell malignancies and multiple myeloma. These include CD19-targeted agents (Yescarta, Kymriah, Tecartus, Breyanzi) and BCMA-directed therapies (Abecma, Carvykti) (41–44). While CAR-T therapy has achieved notable clinical success in hematologic cancers, its efficacy in solid tumors remains limited by three primary obstacles: (1) the immunosuppressive heterogeneity of the tumor microenvironment, (2) antigen loss or downregulation (antigen escape), and (3) insufficient trafficking and infiltration of engineered T cells into tumor sites. To advance therapeutic outcomes, dedicated research efforts must prioritize strategies tailored to overcome the biological and immunological complexities inherent to solid tumors (45–48). Current clinical immunotherapies predominantly target T cells, though their effector mechanisms remain functionally dependent on innate immune interactions to achieve activation, persistence, and immunological memory formation (49). Innate immune components—including NK cells and macrophages—not only prime adaptive immune responses but also exhibit direct tumoricidal activity through cytotoxic signaling and phagocytic clearance. Additionally, innate immune cells expressing Fc receptors (FcRs) amplify adaptive antitumor immunity by facilitating antibody-dependent cellular cytotoxicity (ADCC) and antibody-dependent cellular phagocytosis (ADCP). Given their central involvement in the cancer-immunity cycle, strategically engaging innate immune pathways represents a promising approach to augment therapeutic efficacy and counteract resistance mechanisms. Emerging evidence from preclinical and clinical investigations highlights the potential of CAR-engineered immune cells—spanning conventional T cells, γδ T cells, NK cells, NKT cells, and macrophages—to elicit durable antitumor activity (50–52). Notably, emerging research demonstrates that CAR-modified innate immune cells, mirroring the success of CAR-T therapies, exhibit comparable versatility and therapeutic promise in adoptive cell transfer paradigms (28, 53–55).

## 3 CAR structure

**Figure 1:**
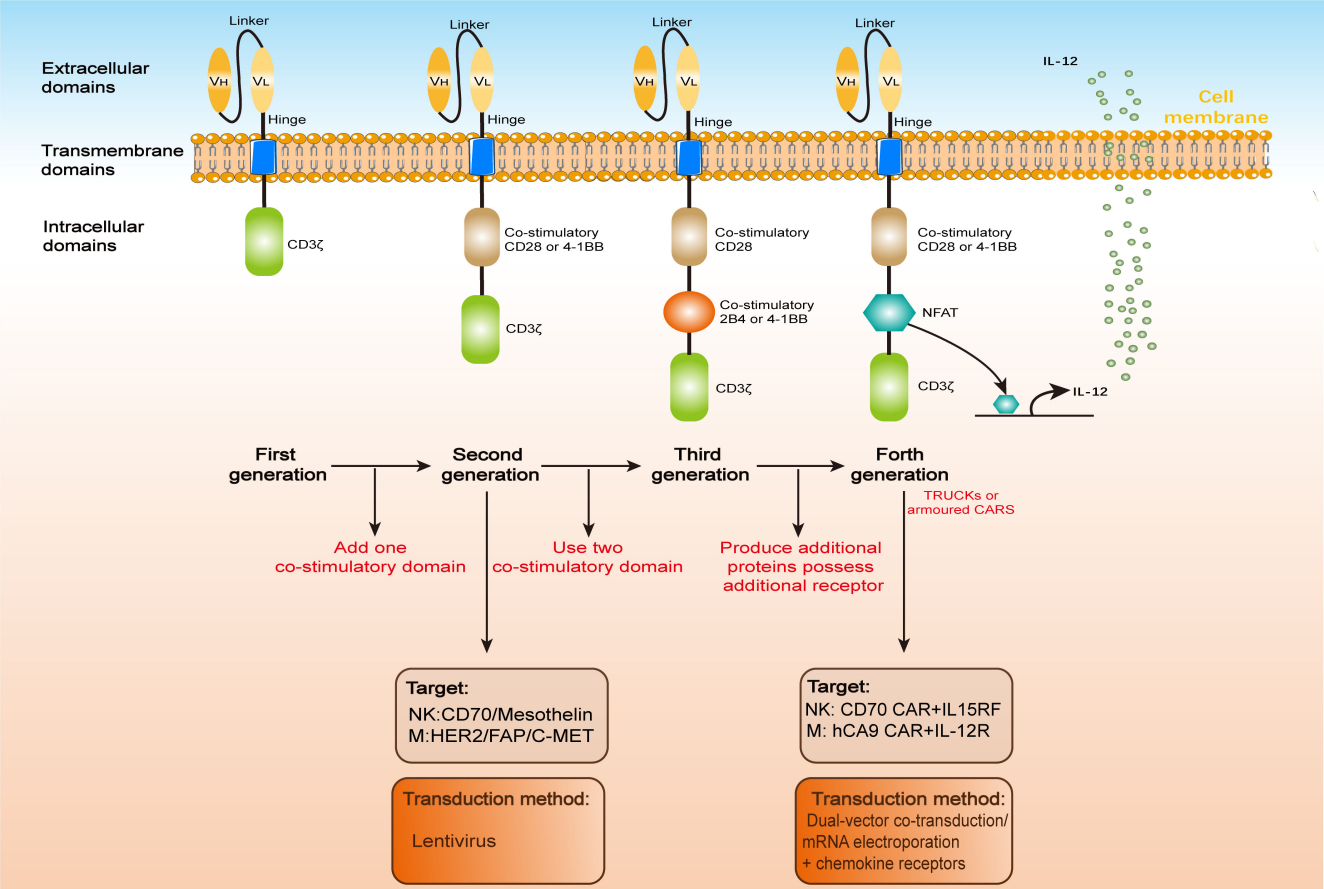
CAR architecture. CARs, whether engineered in T cells (CAR-T), natural killer cells (CAR-NK), or macrophages (CAR-Ms), share a conserved modular structure comprising three functional domains: an extracellular antigen-binding region, a transmembrane anchor, and an intracellular signaling module. Over four iterative generations, CAR designs have evolved to enhance therapeutic potency. The extracellular domain typically features a scFv, formed by a variable heavy (VH) and light (VL) chain linked via a flexible peptide spacer. This scFv is anchored to the transmembrane domain via a hinge region, enabling antigen recognition. Upon target engagement, the intracellular domain transduces activation signals to the host immune cell. First-generation CARs, limited by a solitary CD3ζ signaling motif, exhibit suboptimal antitumor efficacy compared to second-generation constructs incorporating co-stimulatory domains (e.g., CD28 or 4-1BB). Third-generation CARs further amplify signaling by integrating dual co-stimulatory molecules (e.g., CD28-41BB or CD28-OX40), while fourth-generation “armored” CARs (e.g., TRUCKs) augment functionality through inducible cytokine secretion or auxiliary receptor expression.

CARs are synthetic receptors comprising an extracellular single-chain variable fragment (scFv) for antigen recognition (56), a hinge and transmembrane domain for structural stability, and intracellular signaling domains (e.g., CD3ζ) for T-cell activation. Second-generation and later CARs incorporate costimulatory domains (e.g., CD28, 4-1BB) to enhance potency and persistence. “Armored” CARs include additional modifications to improve safety and efficacy against solid tumors (57, 58). We have summarized the updates and iterations of CAR used in immune cell (**Figure 1**).

Most CAR-NK cell research has employed CAR constructs mirroring those utilized in CAR-T cell studies. These constructs either feature the same CD3ζ intracellular domain found in first-generation CAR-T cells (59) or incorporate the CD3ζ along with the co-stimulatory domain 4-1BB, characteristic of second-generation CAR-T cells (60). The incorporation of the 4-1BB co-stimulatory domain notably enhances NK cell functions, including activation, cytotoxicity, and the secretion of cytokines like interferon-γ and granulocyte-macrophage colony-stimulating factor. Similar to CAR-T cell, each CAR construct is comprised of an antigen recognition module (either immunoreceptor tyrosine-based activation motifs [ITAM] or natural receptors like natural killer group 2 member D [NKG2D]), a transmembrane region (CD8α or CD28), and signaling domains specifical for NK cells (such as 2B4, DNAX activation protein of 10 kDa [DAP10], or DNAX activation protein of 12 kDa [DAP12]). The selection of the targeted antigen is pivotal in the design of CARs, encompassing antigens such as CD19 and HER2. The scope of targeted tumor cells primarily encompasses hematological malignancies (including B-cell malignancies targeted by CD19) and solid tumors (such as epidermal growth factor receptor [EGFR] or programmed cell death ligand 1[PD-L1]-positive tumors). Given the intricate balance of activating and inhibitory receptors that regulate NK cell activation and cytotoxicity, the incorporation of NK-specific intracellular signaling domains—such as the adapter molecule DAP10 or ITAM-containing domains like DAP12 and 2B4, have been proposed to enhance cytotoxicity potentially.

2B4, a surface receptor belonging to the signaling lymphocytic activation molecule (SLAM) family, propagates activating signals in NK cells through its adaptor protein SLAM-associated protein (SAP) (60). The interaction between 2B4 and its ligand CD48 on target cells initiates NK cell activation. This engagement subsequently amplifies cytotoxic activity and stimulates IFN-γ secretion. Critically, CAR-NK cells engineered with 2B4-CD3ζ signaling domains demonstrate superior functionality compared to 4-1BB-CD3ζ-based constructs, exhibiting enhanced cytotoxicity, IFN-γ production, and *in vivo* tumor control (61). To refine CAR-NK design paradigms, Li et al. systematically compared a CAR-T configuration with nine CAR-NK variants featuring four transmembrane domains and diverse signaling modules targeting mesothelin (62). Among evaluated configurations, CAR-NK cells engineered with an NKG2D transmembrane domain, a 2B4 co-stimulatory domain, and a CD3ζ signaling module demonstrated robust antigen-specific cytotoxicity. Notably, human induced pluripotent stem cell (iPSC)-derived CAR-NK cells harboring this design retained prototypical NK cell surface markers, displayed sustained *in vivo* survival, and elicited potent antitumor responses in preclinical models. A critical challenge in CAR-NK therapy lies in prolonging their persistence within peripheral circulation and tumor-infiltrating compartments. To address this limitation, researchers have integrated cytokine transgenes—such as IL-21, IL-15, IL-7, or IL-2—into CAR constructs to enhance NK cell proliferation and survival (63). Complementary strategies employ feeder systems, including autologous PBMCs, Epstein-Barr virus (EBV)-transformed lymphoblastoid cell lines (LCLs), or NK-sensitive cell lines (e.g., K562, 721.221), to sustain post-infusion expansion (64, 65). Müller et al. further demonstrated that dual engineering of NK cells with an EGFR vIII-targeted CAR and the CXC chemokine receptor 4 (CXCR4) augmented tumor infiltration capacity, thereby improving therapeutic outcomes in solid tumor models (66).

Similarly, most CAR-M cell research has employed CAR constructs that mirror those used in CAR-T or CAR-NK cell studies. These constructs share either the same CD3ζ intracellular domain as first-generation CAR-T cells (67), or integrate the CD3ζ with the co-stimulatory domain 4-1BB, typical of second-generation CAR-T cells (29). Additionally, they incorporate macrophage-specific intracellular signaling domains, such as Toll/IL-1R (TIR), which promotes the polarization of macrophages towards the M1 phenotype. Notably, TIR has demonstrated the highest efficiency when utilized as the intracellular signaling domain (28).

First-generation CAR-Ms, designed with CD3ζ signaling domains, demonstrated antigen-specific phagocytic activity against tumor cells. Building on this foundation, Lei et al. engineered induced pluripotent stem cell-derived macrophages (iMACs) expressing a CAR incorporating the Toll-like receptor 4 (TLR4)-derived TIR domain. These TIR-integrated CAR-Ms exhibited markedly enhanced antitumor efficacy compared to CAR-M1 constructs (28). Further refinement led to a tandem CD3ζ-TIR dual-signaling CAR, which endowed iMACs with multifunctional capabilities: (1) targeted phagocytosis, (2) antigen-dependent polarization toward pro-inflammatory M1 phenotypes, (3) resistance to immunosuppressive M2 reprogramming, and (4) TME modulation. Beyond core CAR components, emerging strategies propose integrating inflammatory signaling modules (e.g., cytokine receptors or pattern recognition receptors) into CAR architectures. Such innovations aim to convert immunologically inert (“cold”) tumors into inflamed (“hot”) microenvironments, thereby sensitizing malignancies to immune surveillance (68). This genetic modification induced a significant enhancement in phagocytic activity and improved therapeutic efficacy of CAR-Ms. Parallel investigations revealed that integrating TLR4 and/or IFN-γ receptor signaling modules into CAR architectures stimulated macrophages to upregulate M1-polarization markers—including CD86, MHC class II (MHC-II), and TNF-α—while accelerating tumor regression in preclinical models (69). Analogous results emerged in macrophages transfected with sequential intracellular domains of CD3ζ and IFN-γ receptors, or CD3ζ alone, confirming the critical role of pro-inflammatory signaling in optimizing CAR-M functionality (70). Recently, a second-generation CAR-M, derived from iPSCs and engineered with a hybrid CD3ζ-TLR4 intracellular domain, demonstrated superior tumoricidal activity and TME remodeling capabilities compared to earlier iterations (71). Zhang et al. developed HER2-targeted chimeric antigen receptor macrophages (CAR-147) comprising a scFv fused to a hinge region, CD147 transmembrane domain, and intracellular signaling module. These CAR-Ms selectively upregulated matrix metalloproteinases (MMPs), including MMP9, MMP10, and MMP12, facilitating extracellular matrix (ECM) degradation in tumors (72–74). Notably, the CAR-CD147 design achieved ECM disruption without compromising phagocytic capacity, inflammatory cytokine secretion, or reactive oxygen species (ROS) production. Furthermore, this construct significantly inhibited tumor progression and enhanced intratumoral T-cell infiltration *in vivo* (72, 73). In a parallel approach, Niu et al. engineered CAR-Ms to express C-C motif chemokine ligand 19 (CCL19), the natural ligand for C-C motif chemokine receptor (CCR7), to chemoattract CCR7+ immunosuppressive cells. This strategy promoted CD3+ T-cell recruitment into tumors, amplified pro-inflammatory cytokine levels (e.g., IFN-γ, TNF-α), suppressed tumor growth and metastasis, and prolonged survival in preclinical models (75).

Apart from designing the CAR, the preparation of the cell source for loading the CAR cells, and the functionality of the loaded CAR cells, are both crucial steps in the process.

## 4 Beyond CAR-T: next-generation CAR-engineered cell therapies

To address the limitations of CAR-T cells in solid tumors, research has expanded to harness other immune effector cells, each offering distinct biological advantages.

### 4.1 CAR-NK cells: innate recognition and safety

#### 4.1.1 Cell source

NK cells derive from CD34+ hematopoietic progenitor cells (HPCs) primarily located in the bone marrow. Their development involves sequential, tightly regulated differentiation stages: CD34+ HPCs first commit to lymphoid lineage by transitioning into lymphoid-primed multipotent progenitors (LMPPs). LMPPs subsequently differentiate into common lymphoid progenitors (CLPs), which further specialize into NK cell precursors (NKPs). These NKPs undergo terminal maturation to yield functionally competent mature NK cells (mNKs). While bone marrow remains the principal site of NK cell ontogeny, extramedullary pathways contribute to NK cell heterogeneity. Early lymphoid precursors (ELPs) within the thymus exhibit bipotent potential, capable of generating both T cells and NKPs under specific cytokine milieus. Furthermore, NKPs have been identified in peripheral tissues, including the liver, lymph nodes, and spleen. These tissue-resident NKPs undergo localized maturation, producing functional NK cells that adapt to regional immunological demands (76, 77).

**Figure 2:**
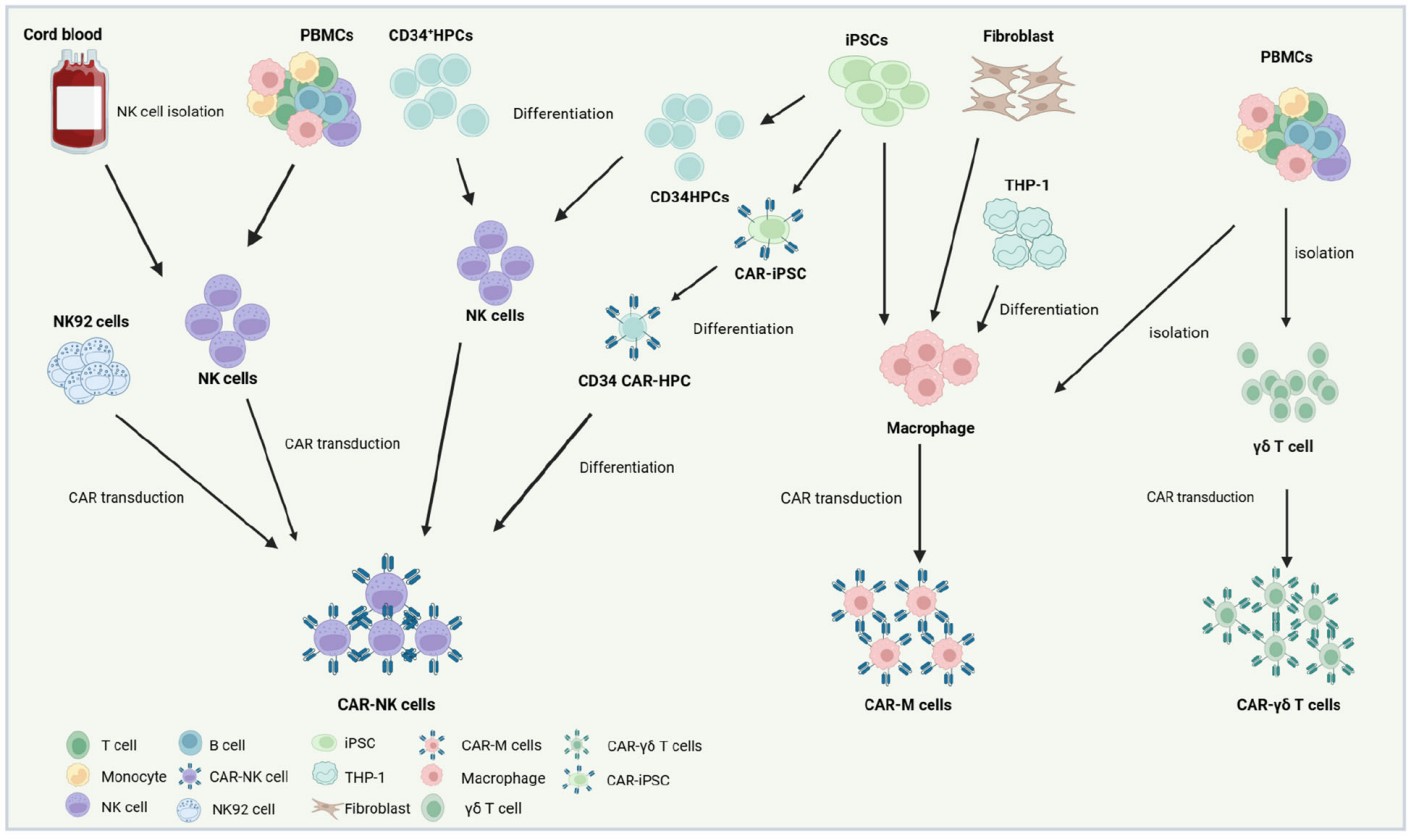
Production strategies and cellular sources for CAR-NK,CAR-M and CAR-γδ T therapeutics. The NK92 cell line represents a widely utilized source for CAR-NK manufacturing due to its capacity for indefinite *in vitro* expansion and resilience to freeze-thaw cycles. However, as a malignant line derived from natural killer cell lymphoma, CAR-engineered NK92 cells require γ-irradiation prior to clinical infusion to eliminate proliferative risks. Primary NK cells, by contrast, are isolated from PBMCs of healthy donors or UCB via immunomagnetic selection, followed by activation, genetic modification with viral vectors (e.g., lentiviral or retroviral systems), and expansion in cytokine-enriched media to achieve clinical-grade quantities. Alternatively, CD34+ HPCs can be differentiated into NK lineages using defined cytokine cocktails, subsequently engineered with CARs, and amplified *in vitro* for therapeutic use. Recent advances highlight induced pluripotent stem cells (iPSCs) as a scalable platform for “off-the-shelf” CAR-NK production. iPSCs undergo sequential differentiation into CD34+ HPCs and functional NK cells, with CAR integration achievable at the pluripotent stage. This approach enables standardized generation of CAR-iPSC-derived NK cells, circumventing donor variability. For CAR-Ms, primary macrophages are typically isolated from donor PBMCs or differentiated *in vitro* from THP-1 cell lines, fibroblast-derived progenitors, or iPSCs. Following isolation or differentiation, these cells are transfected with CAR-encoding constructs and functionally validated prior to adoptive transfer. iPSC-derived CAR-Ms further offer potential for renewable, genetically uniform therapeutic batches. For CAR-γδ T, primary γδ T are typically isolated from donor PBMCs. Created using BioRender.com.

NK cells for CAR-engineered therapies are derived from diverse cellular sources, broadly categorized into primary cells, immortalized lines, and stem cell progenitors (**Figure 2**). Primary NK cells are typically isolated from peripheral blood (PB) or umbilical cord blood (UCB) of healthy donors via immunomagnetic selection, yielding populations suitable for clinical-scale expansion. Alternatively, iPSCs provide a renewable platform for generating homogeneous, antigen-specific CAR-NK cells with robust proliferative and cytotoxic activity in preclinical models (78–82). The NK92 cell line remains the most clinically utilized source for CAR-NK products due to its indefinite *in vitro* expansion capacity and resilience to cryopreservation cycles, enabling cost-effective, “off-the-shelf” therapeutic manufacturing (83). However, as a malignant line derived from non-Hodgkin’s lymphoma, NK92 cells necessitate γ-irradiation prior to infusion to mitigate tumorigenic risks. This process compromises *in vivo* persistence and functionality, while inherent deficiencies in activating receptors (e.g., CD16, Natural Killer Cell p44-Activating Receptor [NKp44]) further limit their therapeutic efficacy (84). In contrast, primary PB-derived NK cells, isolated from PBMCs using clinical-grade isolation kits, offer physiological relevance and endogenous receptor diversity. These cells are activated with cytokines (e.g., IL-2, IL-15) and expanded under Good Manufacturing Practice (GMP)-compliant conditions, ensuring suitability for adoptive immunotherapies (51). While logistically challenging due to donor variability, PBMC-sourced NK cells (PB-NK) cells circumvent the safety concerns associated with immortalized lines, positioning them as a pragmatic alternative for personalized CAR-NK regimens. PBMC-derived CAR-NK cells, which retain diverse activating receptors, can be infused without γ-irradiation, preserving their capacity for *in vivo* expansion and persistence. PB-NK are predominantly CD56^dim^CD16^+^ subsets (~90% of circulating NK cells), exhibiting a terminally differentiated phenotype marked by augmented cytotoxicity but limited proliferative potential (85, 86). Critically, PB-NK cells circumvent graft-versus-host disease (GvHD) risks, enabling their use across HLA-matched or mismatched donors. This expands donor availability and enhances batch consistency for clinical-scale production (85), UCB represents an alternative NK cell source, leveraging established HLA-typed banks for donor selection. However, umbilical cord blood-derived NK cells (UCB-NK) face logistical hurdles: limited cell yields per unit (<5 × 10^6^ cells/mL) necessitate pooling or ex vivo expansion to achieve therapeutic doses. Phenotypically, UCB-NK cells display immaturity relative to PB-NK counterparts, characterized by reduced expression of cytolytic mediators (perforin, granzyme B), activating receptors (CD16, killer immunoglobulin-like receptors [KIR]), and adhesion molecules, alongside elevated inhibitory natural killer cell group 2, member A (NKG2A) levels (87). These traits correlate with diminished tumoricidal activity *in vitro*, though CAR engineering may partially rescue functionality. Both PBMC- and UCB-derived CAR-NK products are constrained by donor-dependent heterogeneity, complicating therapeutic standardization (88). iPSCs offer a paradigm shift, enabling the generation of homogeneous, genetically tailored CAR-NK cells with uniform receptor profiles and reproducible antitumor responses. iPSC-derived CAR-NKs exhibit scalable production, bypassing donor variability while maintaining clinical-grade potency in preclinical models (88, 89). They provide an unlimited, scalable supply with enhanced consistency, avoiding donor variability and finite expansion issues of primary cells. Unlike immortalized lines (e.g., NK-92), iPSC-derived cells reduce tumorigenic risks and exhibit improved functionality, such as antigen-specific cytotoxicity and metabolic fitness.

#### 4.1.2 The therapeutic mechanism of CAR-NK cells

**Figure 3:**
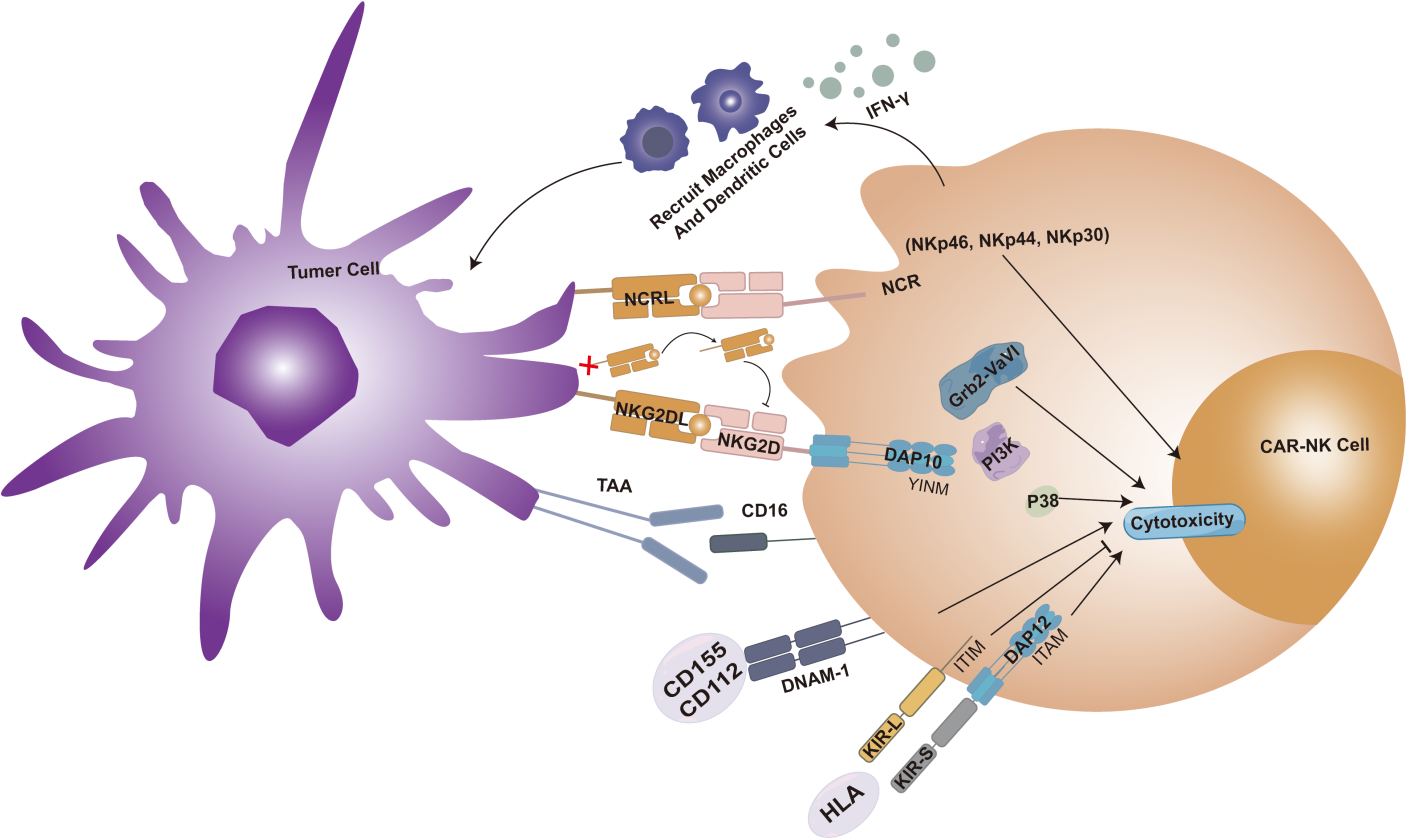
CAR-NK cell cytotoxicity is enhanced when the surface NKG2D receptor binds its ligand (NKG2DL), forming a complex with DAP10 that activates the Grb2-Vav1-P85 and PI3K signaling pathways. Within the tumor microenvironment (TME), cytokines promote NKG2D receptor activation, whereas soluble NKG2DL (sNKG2DL) exerts an inhibitory effect. Killer immunoglobulin-like receptors (KIRs) are classified into activating KIR-S (containing ITAM motifs) and inhibitory KIR-L (containing ITIM motifs) based on cytoplasmic domain sequence differences. Engagement of DNAM-1 with ligands CD155 or CD112 also potentiates CAR-NK cytotoxicity. Additionally, the natural cytotoxicity receptor (NCR) family, comprising activating surface receptors NKp46, NKp44, and NKp30, contributes to CAR-NK cell activity. IFN-γ secretion triggers the recruitment of macrophages and DCs, presenting an advantageous alternative anti-tumor mechanism. Furthermore, NK cells can stimulate ADCC-mediated cancer cell killing by recognizing the Fc fragment of IgG bound to tumor cell epitopes.

NK cells possess significant anti-tumor effects, and their mechanism distinct from T cells, which rely on antigen recognition. The anti-tumor function of NK cells is primarily mediated via the release of perforin and granzymes upon activation. Natural cell toxicity receptors (NCRs) are a group of activating receptors on the surface of natural killer cells, including NKp46, NKp30, and NKp44. NK cells express transmembrane receptors, such as KIRs, NKG2D, and DNAX accessory molecule-1 (DNAM-1), facilitating calreticulin-mediated cancer cell recognition and apoptosis. Among these, NKG2D, an activating receptor, is pivotal in inducing caspase-mediated apoptosis and determines NK cells’ recognition of cancer stem cells (CSCs). When NKG2D binds to stress-induced cellular ligands on tumor cells, like MHC class I chain-related proteins A/B (MICA/B), it can also induce NK cell cytotoxicity (90). About DNAM-1, studies have demonstrated that NK92 cells overexpressing DNAM-1inmprove robust degranulation release and potent anti-sarcoma activity. Similarly, enhanced degranulation has been observed in several other solid tumor cell lines, including prostate, pancreatic, colon, and lung cancer cell lines. Notably, these receptors are also expressed in NKT cells, encompassing both inhibitory and activating receptors. The cytotoxic activity of NK cells is primarily modulated by KIR signaling, with MHC I molecules serving as crucial ligands for KIR receptors. The intricate KIR/MHC relationship is indispensable for NK cell activity and the application of NK cell therapy (91). IFN-γ secretion triggers the recruitment of macrophages and DCs, presenting an advantageous alternative anti-tumor mechanism. Furthermore, NK cells can stimulate ADCC-mediated cancer cell killing by recognizing the Fc fragment of IgG bound to tumor cell epitopes. This recognition is crucial in treating solid tumors with anti-HER2 and anti-EGFR therapies. The efficient triggering of ADCC by NK cells elucidates why, in the context of multiple solid tumors, the infiltration of NK cells within TME is associated with a more favorable prognosis (92, 93). We summarized the cytotoxic effects of CAR-NK cells on tumor cells (**Figure 3**).

CAR-NK cells not only possess robust anti-tumor activity but also markedly augment their lethal impact on tumor cells. Through the transduction of CAR constructs, NK cells could specifically target tumor cells, thereby enhancing the precision and efficacy of our treatment. Although constructing CAR-NK cells using the classic intracellular domains derived from CAR-T cells has been proved effectively (94), a multitude of studies have demonstrated that CAR-NK cells incorporating NK-specific co-stimulatory domains, such as NKG2D, 2B4, DNAM1, DAP-10, or DAP-12, exhibit enhanced cytotoxicity and increased secretion of IFN-γ. These findings have been validated in HCC cell lines, Non-small-cell lung cancer (NSCLC) studies, xenograft OC mouse models, and prostate cancer xenograft mouse models (95–98). By secreting the IL-2Rβγ agonist—Neo-2/15—to optimize CAR-NK cell metabolism, their function within the metabolically impaired TME can be enhanced. This approach offers a potentially universal strategy for modulating NK cell activity against immune suppression in solid tumors through metabolic modification (24).

#### 4.1.3 Challenges to the clinical applications of CAR-NK cells

##### 4.1.3.1 Ex vivo expansion of primary CAR-NK cells

While CAR-NK cells hold significant therapeutic potential for cancer immunotherapy, a critical barrier to their clinical translation lies in achieving robust ex vivo expansion of primary CAR-NK populations. Current methodologies for generating clinical-grade NK cells predominantly rely on PB, UCB, or embryonic stem cell sources, often requiring complex activation protocols (99). Co-culturing with irradiated feeder cells (e.g., K562-mb15-4-1BBL) remains a common strategy for NK cell expansion, yet concerns persist regarding residual feeder cell contamination in final therapeutic products (100). Notably, a clinical trial evaluating IL-15/4-1BBL-activated NK cell infusions (aNK-DLI), expanded via K562 feeder cells, reported Grade 4 GvHD in three patients despite HLA matching and T cell depletion (101). This suggests feeder cell-derived factors or residual contaminants may inadvertently trigger alloreactive responses, underscoring safety risks inherent to feeder-dependent systems. To mitigate these challenges, feeder-free expansion protocols have gained traction. Cytokine-driven approaches, such as IL-2 or IL-15 supplementation combined with anti-CD3 monoclonal antibodies, enable NK cell proliferation directly from PBMCs while minimizing exogenous contamination (102). Masuyama et al. recently advanced this paradigm by developing a novel protocol for high-purity NK cell generation (103). PBMCs are co-stimulated with anti-CD3 and anti-CD52 monoclonal antibodies, then cultured for 14 days in NKGM-1 medium supplemented with autologous plasma and IL-2. This method achieves approximately 60% NK cell purity after 7 days, with further enrichment upon extended culture, yielding a median of 5.7 × 10^9^ NK cells from 20 mL of PB—a 646-fold expansion within 14 days. Such feeder-free strategies not only enhance safety profiles but also improve scalability, addressing critical bottlenecks in CAR-NK manufacturing. Future efforts must balance expansion efficiency with functional fidelity to ensure therapeutic efficacy in clinical settings.

Spanholtz et al. established a clinically scalable, feeder-free protocol for ex vivo expansion of NK cells from UCB-derived CD34+ HPCs. Their two-step differentiation strategy employed a serum-free, clinical-grade medium supplemented with a cytokine cocktail—stem cell factor (SCF), IL-7, FMS-like tyrosine kinase 3 ligand (Flt3L), thrombopoietin (TPO), IL-15, granulocyte colony-stimulating factor (G-CSF), granulocyte-macrophage CSF (GM-CSF), IL-6, and IL-2. This approach achieved a mean expansion exceeding 15,000-fold, yielding near-pure (>99%) CD56^+^CD3^−^ NK cell populations. Functionally, UCB-NK cells demonstrated potent cytotoxicity against myeloid leukemia (K562) and melanoma (A375) cell lines, validating their therapeutic potential (104). In parallel, Lupo et al. developed an alternative feeder- and stroma-free platform for generating iPSC-NKs (105). By leveraging centrally authenticated iPSC lines, the authors circumvented protocol-dependent variability inherent to donor-specific reprogramming. Differentiation omitted peripheral blood components and TrypLE dissociation, enhancing reproducibility. The resulting iPSC-NKs exhibited robust antitumor activity, characterized by elevated cytokine secretion (e.g., IFN-γ), degranulation markers (CD107a), and cytotoxicity against solid tumor lines (e.g., ovarian SKOV3) and patient-derived xenograft models. This standardized methodology highlights iPSC-NKs as a scalable, donor-agnostic alternative to primary NK sources.

##### 4.1.3.2 CAR transduction into NK cells

A critical limitation in CAR-NK cell manufacturing lies in ensuring robust, durable CAR expression to sustain therapeutic efficacy. Advances in genetic engineering have enabled diverse methodologies for CAR integration, broadly categorized into viral and non-viral strategies. Viral approaches, primarily utilizing lentiviral or γ-retroviral vectors, achieve stable genomic integration through reverse transcription, enabling long-term CAR expression. However, these systems face challenges such as insertional mutagenesis risks, limited cargo capacity, and cytotoxicity during transduction. Non-viral alternatives prioritize safety and scalability. Plasmid-based transfection, though cost-effective, suffers from low efficiency in primary NK cells. Transposase-mediated systems (e.g., Sleeping Beauty or PiggyBac) enable site-specific integration without viral components, yet require optimization for NK cell compatibility. mRNA electroporation provides transient CAR expression, reducing genotoxicity risks while allowing dose-controlled activity—a feature advantageous for mitigating cytokine release syndrome. CRISPR-Cas9 or TALEN-mediated genome editing further refines precision, enabling knock-in CAR insertion at safe genomic loci (e.g., TRAC), though delivery efficiency remains a hurdle in non-dividing NK cells. Emerging hybrid strategies, such as nanoparticle-encapsulated mRNA or transposon-plasmid complexes, aim to balance persistence and safety. Functional validation studies emphasize the need for context-specific optimization: viral vectors for durable solid tumor engagement versus mRNA for acute, controlled responses in hematological malignancies (106).

Viral vectors, particularly retroviral and lentiviral systems, remain the predominant tools for stable genomic integration of CARs into primary NK cells. Retroviral vectors achieve high transduction efficiencies in *ex vivo*-expanded NK cells (60), yet their propensity for random genomic integration raises concerns about insertional mutagenesis and oncogenic transformation. For example, a clinical trial utilizing retroviral gene therapy reported T-cell leukemia development in 4 of 9 patients, underscoring these risks (107). In contrast, lentiviral vectors exhibit a safer integration profile but demonstrate variable transduction efficacy dependent on the NK cell source: PB-derived NK cells show modest efficiencies (8–16%), while UCB-sourced NK cells achieve markedly higher rates (~73%) (108). This disparity positions UCB-NK cells as a superior substrate for CAR engineering. To enhance lentiviral transduction in refractory NK populations, researchers have incorporated viral entry facilitators. Polybrene, a cationic polymer that neutralizes electrostatic repulsion between viral particles and cell membranes, improves transduction by ~30% (109). Retronectin, a recombinant fibronectin fragment, enhances viral tethering to cell surfaces via heparan sulfate proteoglycan binding, increasing transduction rates by 2–3 fold (110) Vectofusin-1, a synthetic cationic peptide, further optimizes this process by promoting viral fusion with NK cell membranes, achieving up to 80% efficiency in clinical-grade CAR-NK production (111). Collectively, these innovations have refined viral-based CAR-NK engineering, balancing efficacy with mitigated genotoxic risks.

Despite the elevated transduction rates associated with viral vector-based CAR-T cell manufacturing, these methods remain constrained by prohibitive costs and risks linked to stochastic genomic integration. Consequently, recent efforts have prioritized non-viral strategies for engineering CAR-NK cells, which offer improved safety and scalability. Plasmid-based systems, while cost-effective and minimally immunogenic, are limited by transient transgene expression due to episomal DNA retention. In contrast, the Sleeping Beauty (SB) transposon system—a non-viral gene transfer platform—enables stable genomic integration, combining the durability of viral vectors with reduced genotoxicity risks. The SB system operates via a binary vector design: (1) a transposon carrying the CAR transgene, flanked by inverted terminal repeats (ITRs), and (2) a transposase enzyme (e.g., hyperactive SB100X variant) that catalyzes “cut-and-paste” integration of the transposon into TA-rich genomic loci. This platform has been successfully deployed in T cells, where electroporation-mediated delivery of SB components yields stable CAR expression with clinical efficacy (112–114). However, translating this approach to primary NK cells faces challenges, including low transfection efficiency (<20% in unstimulated NKs) and cytotoxicity from electroporation-induced membrane damage. Emerging evidence suggests potential for SB in NK cell engineering. The system has demonstrated stable gene transfer in umbilical cord blood-derived hematopoietic stem/progenitor cells (HSPCs) (114), and a follow-up study validated SB-mediated anti-CD19 CAR expression in HSPC-derived NK cells with tumoricidal activity (115). These findings highlight the SB system’s adaptability, though optimization of delivery methods (e.g., nanoparticle encapsulation) is critical to overcome NK-specific barriers.

The transient delivery of CAR-encoding mRNA via electroporation has emerged as a promising strategy for engineering human primary NK cells. This approach offers distinct advantages, including rapid production timelines, cost efficiency, and minimized genotoxicity risks due to the absence of genomic integration. Shimasaki et al. demonstrated that electroporation of anti-CD19 CAR mRNA achieved robust transient expression, with 61.3% of NK cells exhibiting CAR positivity and 90% viability at 24 hours post-transfection (116). Notably, mRNA electroporation achieves high transfection efficiencies (80–90%) in both *in vitro*-expanded NK cells and primary resting NK populations, even without cytokine preactivation. However, the therapeutic utility of this method is constrained by the transient nature of CAR expression, which typically diminishes within 3 days (117). Consequently, mRNA-electroporated CAR-NK cells may be most effective as adjuvant therapies to induce rapid tumor debulking, rather than durable remission. Their short-lived activity could synergize with conventional treatments (e.g., chemotherapy) to reduce initial tumor burden, while mitigating risks of prolonged immune activation. Further optimization of mRNA stability or repeat dosing regimens may extend functional persistence for broader clinical applications.

The CRISPR/Cas9 system, a revolutionary gene-editing tool, operates through guide RNA (gRNA)-directed Cas nuclease activity to induce sequence-specific double-strand DNA breaks, enabling precise genomic modifications (118). Leveraging this technology, Alexander G. Allen et al. demonstrated the utility of the *SLEEK* (Safe Landing Site Exclusively Engineered Kinase) platform to enhance the therapeutic potential of iPSC- NK cells. By integrating CD16 (FcγRIIIa) and membrane-bound interleukin-15 (*mbIL-15*) into the GAPDH locus—a constitutively active genomic safe harbor—the researchers generated SLEEK double knock-in (DKI) iPSCs. These engineered iPSCs were subsequently differentiated into iPSC-NK (iNK) cells, which exhibited enhanced ADCC via CD16 and prolonged persistence due to mbIL-15 signaling. In preclinical models, SLEEK DKI iNK cells demonstrated substantial enhancement in tumor-killing efficacy and survival *in vitro* and *in vivo* compared to unmodified counterparts (119). In parallel, retroviral vector-based strategies have enabled dual genetic engineering of primary NK cells. A recent study co-delivered Cas9-sgRNA complexes and anti-EGFR CAR transgenes via retroviral particles, simultaneously introducing CAR expression and disrupting the TIGIT gene—an inhibitory checkpoint receptor. This approach achieved efficient CAR integration (≥70%) and TIGIT knockout (≥85%), yielding CAR-NK cells with augmented cytotoxicity and resistance to tumor-mediated immunosuppression. The dual-editing strategy highlights CRISPR’s versatility in overcoming intrinsic NK cell limitations while retaining viral transduction efficiency for clinical scalability (120).

### 4.2 CAR-M: phagocytosis and TME remodeling

Unlike natural killer cells, macrophages possess a separate set of functions in the following areas.

#### 4.2.1 Cell source

Macrophages are monocytes derived from hematopoietic stem cells in the bone marrow, which further mature within tissues. These monocytes circulate in the bloodstream and migrate to different tissues and organs as needed, developing into macrophages with specific functions. The development and differentiation of macrophages are regulated by various cytokines and signals, which can influence their morphology, functions, and lifespan (121–123). Macrophages play a crucial role in the immune system. They are capable of phagocytosing and digesting pathogens, clearing senescent and damaged cells, and secreting a multitude of cytokines to regulate immune responses. Furthermore, macrophages are also involved in tissue repair and regeneration processes.

Macrophages can be originate from various origins, including the PB of healthy donors (124), iPSCs (28), fibroblasts (125), and commercially accessible mouse cell lines like RAW264.7 and J774A.1, as well as human cell lines such as THP-1 (124, 126) (**Figure 2**). Immortalized murine cell lines and human monocytic lines serve as reproducible platforms for preclinical CAR-M development, demonstrating potent phagocytic activity against tumor targets *in vitro* and *in vivo* (127, 128). Utilizing macrophages harvested from the ascitic fluid of cancer patients as a source for CAR-M production represents a practical approach that supports clinical treatment (129). Stem cell-derived macrophages represent a scalable alternative. iPSCs and UCB-derived hematopoietic progenitors can be differentiated into functional CAR-Ms that exhibit antigen-specific cytokine secretion, pro-inflammatory polarization (M1 phenotype), and tumor cell phagocytosis, iPSC-based systems, in particular, enable standardized, large-scale CAR-M generation, circumventing the variability and low yields associated with primary PBMC isolation. Despite these advances, industrial-scale CAR-M manufacturing faces challenges. Primary PBMC-derived macrophages are limited by donor variability and batch inconsistencies, while cell line-based models may lack physiological relevance. Integrating stem cell differentiation protocols with bioreactor technologies offers a promising solution, ensuring consistent production of clinical-grade CAR-Ms with stable functional profiles. This approach aligns with the growing demand for “off-the-shelf” immunotherapies capable of overcoming logistical and economic barriers in cancer treatment (28, 130, 131).

Immune cells derived from iPSCs theoretically have an advantage in addressing challenges due to their flexibility for expansion and gene editing at the iPSC stage. For example, CAR-T cells differentiated from iPSCs have been shown to be effective in preclinical studies for treating B-cell cancer cells (132), CAR-NK cells for treating kidney cancer cells (133), and CAR-M cells for treating ovarian cancer cells (131). CAR-Ms can specifically “phagocytose” tumor cells or alter the TME through antigen-dependent mechanisms, providing tools for direct phagocytosis or regulation of the specific microenvironment at the interface between tumors and immune cells. CAR-modified iPSC-derived myeloid cells may offer a novel off-the-shelf macrophage source with antigen-specific phagocytic capacity and the potential for large-scale production as a standardized cell product. Therefore, iPSC-derived CAR-M represents an engineering-friendly and scalable macrophage platform, serving as an important complement to other iPSC-derived immune cells in cancer immunotherapy.

#### 4.2.2 The therapeutic mechanism of CAR-M cells

**Figure 4:**
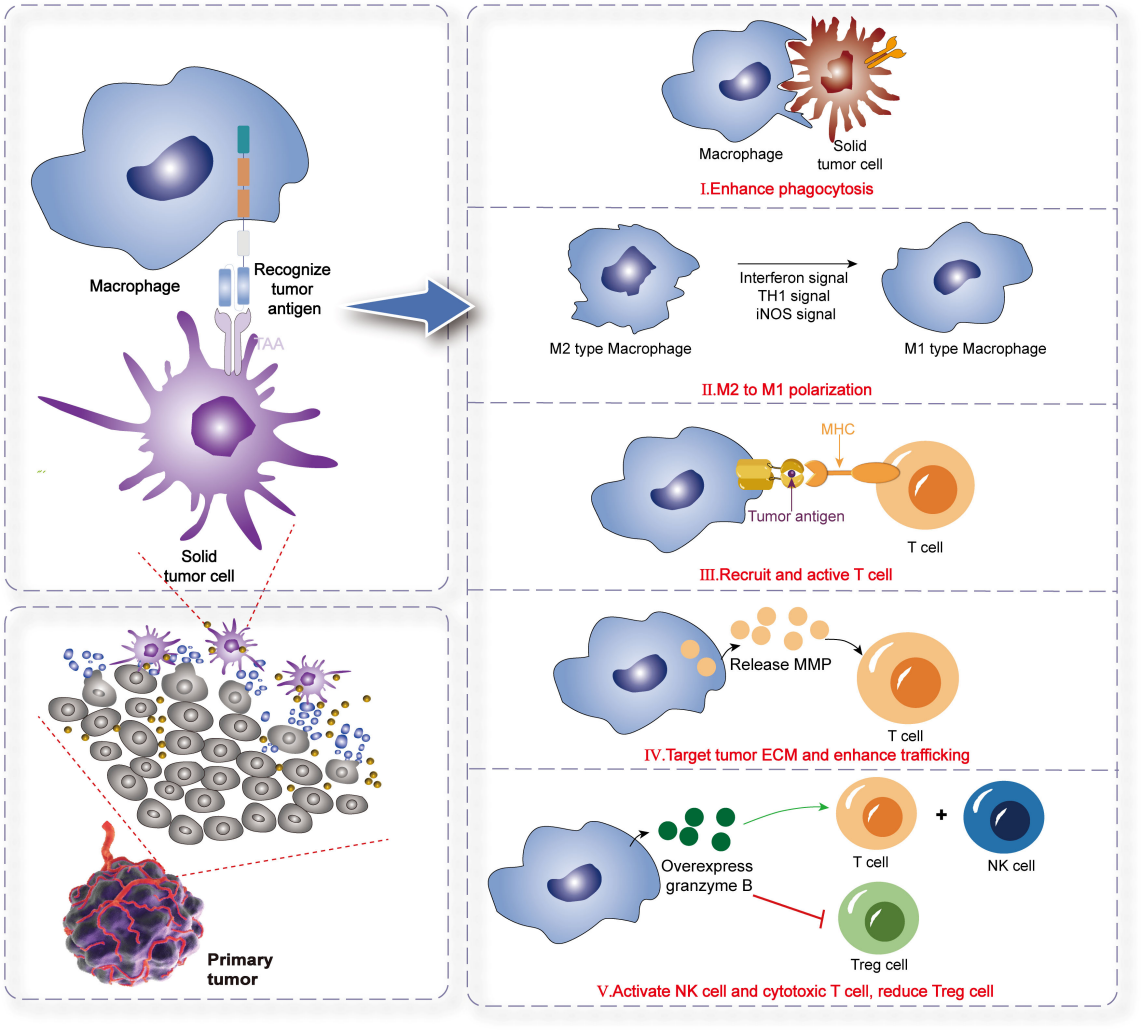
The mechanism of CAR-M therapy for solid tumors. Phagocytosis: CAR-M cells directly engulf and destroy tumor cells via engineered CARs targeting tumor-associated antigens. Antigen Presentation: CAR-M cells process and present tumor antigens via MHC molecules, activating adaptive immune responses (e.g., T cells).Immune Effector Cell Recruitment: They secrete chemokines (e.g., CCL5, IFN-γ) to recruit endogenous T/NK cells, fostering a pro-inflammatory TME. M1 Polarization: CAR-M promotes macrophage polarization toward the pro-inflammatory M1 phenotype (anti-tumor), over immunosuppressive M2, enhancing tumoricidal activity. ECM Remodeling & Enhanced Infiltration: CAR-M releases MMPs to degrade tumor extracellular matrix (ECM), overcoming physical barriers and improving immune cell trafficking into tumors.

Macrophages constitute the most adaptable cell type within the hematopoietic system, executing vital regulatory functions in development, homeostasis, tissue repair, and immunity. These functions are influenced by various factors, including disease state, tissue location, and cellular origin (134). In response to diverse microenvironments and signaling cues, macrophages display a spectrum of phenotypes, primarily manifesting as M1 or M2 activation states. Specifically, M2 macrophages, also termed alternatively activated macrophages, exhibit the capacity to repair tissue damage and induce neovascularization. Cytokines secreted by tumor tissues, such as IL-6 and transforming growth factor (TGF)-β, combined with hypoxic conditions, can mislead macrophages into initiating repair processes and neovascularization within tumors, inadvertently facilitating cancer progression (135, 136). Beyond their well-known phagocytic capabilities, macrophages’ remarkable plasticity and polarization deserve special attention. Diversity and plasticity are defining characteristics of monocytes and macrophages within the mononuclear phagocyte lineage (137). Macrophages in different tissues develop unique phenotypes in response to specific microenvironmental stimuli and signals, potentially undergoing M1 or M2 activation. In the context of immune responses, macrophage polarization mirrors the Type 1 T helper cell (Th1)- Type 2 T helper cell (Th2) polarization of T cells. Notably, macrophages polarized towards the M1 or M2 phenotype can undergo a certain degree of phenotypic reversal. In summary, the M1 phenotype is distinguished by the high expression of inflammatory cytokines and exhibits potent antibacterial and antitumor activities. Conversely, M2 macrophages promote tissue remodeling and, paradoxically, facilitate tumor progression (138). Furthermore, the reversibility of macrophage polarization towards the M1 or M2 phenotype plays a pivotal role in cancer therapy, offering potential therapeutic targets and strategies (139).We summarized the mechanism of CAR-M in eliminating tumors (**Figure 4**).

Equipping human macrophages with specific CARs enhances their phagocytic activity and antigen-presenting capabilities within tumors (140). The fundamental mechanisms underlying the antitumor activity of CAR-M against solid tumors can be outlined as follows: CAR-M cells naturally navigate toward solid tumors, leveraging the innate tumor-homing characteristics of myeloid cells. Through the CAR, CAR-M binds to specific antigens located on the tumor surface, activating its activation. Once activated, CAR-M secretes TNF-α, a cytokine that triggers apoptosis in tumor cells. Following activation, CAR-M secretes inflammatory cytokines, which stimulate the activation of T cells and promote the M1 polarization of tumor-associated macrophages. CAR-M cells specifically target and phagocytose tumor cells. CAR-M presents tumor antigens to the immune system, thereby facilitating the development of adaptive immunity (97).

In light of the limitations associated with CAR-T and CAR-NK cell therapies, CAR-Ms have emerged as a promising alternative for solid tumor immunotherapy. Structurally homologous to CAR-T and CAR-NK cells, CAR-Ms comprise three core components: (1) an extracellular antigen-binding domain targeting tumor-associated antigens (TAAs), (2) a transmembrane anchoring region, and (3) an intracellular signaling module. Current research prioritizes the optimization of intracellular domains to amplify phagocytic activation, with HER2-targeted constructs dominating clinical exploration (NCT04660929). CAR-Ms exhibit unique therapeutic advantages over CAR-T cells, particularly in overcoming barriers posed by the immunosuppressive TME. Unlike T cells, which face stromal-imposed physical exclusion from tumor niches, macrophages inherently infiltrate TMEs via chemotactic gradients. Furthermore, CAR-Ms counteract protumorigenic TAMs—key mediators of immunosuppression and metastasis—by reducing TAM abundance and repolarizing residual populations toward antitumor phenotypes. Beyond direct phagocytosis, CAR-Ms enhance adaptive immunity through antigen cross-presentation and CD8+ T cell priming, while their shorter *in vivo* persistence reduces off-tumor toxicity risks. Hypoxia-responsive migratory capacity further enables CAR-Ms to penetrate avascular tumor regions, leveraging metabolic cues for targeted accumulation. Despite these advantages, CAR-M therapeutics remain in an early developmental phase. Key challenges include optimizing CAR-M durability, cryopreservation protocols, and scalable manufacturing. Transient persistence necessitates repeated dosing to sustain therapeutic activity, raising logistical and cost concerns. Additionally, CAR-M functional attenuation within TMEs—driven by checkpoint ligand upregulation and metabolic competition—requires combinatorial strategies to maintain cytotoxic potency.

#### 4.2.3 Strategies to overcome the limitations in CAR-M bioengineering

Significant progress in genetic engineering methodologies has driven the development of diverse viral and non-viral strategies to enhance gene delivery efficiency in immune effector cells. For myeloid cell modification, lentiviral vectors incorporating Vpx—an accessory protein that counteracts host restriction factors—have demonstrated superior transgene delivery to macrophages, bypassing intracellular barriers (141). An alternative approach employs the chimeric adenoviral vector Ad5f35, which achieves high-efficiency transduction in primary human macrophages due to its tropism for the CD46 receptor (142). Notably, Ad5f35-engineered macrophages not only sustain transgene expression but also retain proinflammatory M1 polarization, a phenotype stabilized through NLRP3 inflammasome activation triggered by adenoviral DNA sensing (143). In parallel, non-viral platforms such as Sleeping Beauty transposon systems, mRNA electroporation, and plasmid DNA transfection have emerged as scalable alternatives for macrophage bioengineering (144–146). Kang et al. advanced this field by developing mannose-conjugated polyethyleneimine (MPEI) polymer nanoparticles to co-deliver CAR constructs and IFN-γ mRNA, synergistically enhancing macrophage phagocytic capacity and anti-tumor activity (70).

Despite these innovations, non-viral CAR delivery remains challenging due to macrophage resistance to exogenous nucleic acid uptake. Recent efforts prioritize lipid-based nanotechnologies to circumvent these limitations. Lipid nanoparticles (LNPs), optimized for biocompatibility and endosomal escape, have enabled efficient CAR mRNA delivery (147, 148). Ye et al. pioneered LNP formulations encapsulating CD19-targeted CAR mRNA, achieving functional expression in both murine macrophages and human T cells (149). Through systematic screening, phospholipid integration was identified as critical for stabilizing mRNA-LNP complexes, while codon optimization and nucleoside modification enhanced translational fidelity and cytotoxicity against B-cell malignancies. These findings underscore LNPs as a versatile platform for immune cell engineering, with implications for modular CAR therapeutic design. NKG2D-CAR-expressing macrophages significantly enhance their ability to remodel the tumor microenvironment and eliminate gliomas after co-expression of IL-12 and IFNα2 (150).

### 4.3 The therapeutic application of γδ T cells in oncology

The ACT, particularly with CAR T cells, has inaugurated a new era in oncology. While αβ T cell-based products have demonstrated profound efficacy in hematological malignancies, their clinical utility is constrained by MHC restriction, the risk of GvHD, and suboptimal performance against solid tumors (151). This has prompted an intensive search for alternative cellular platforms. γδ T cells, a distinct lymphocyte population with pleiotropic functions bridging innate and adaptive immunity, have emerged as a particularly promising candidate (152). Their qualities of MHC-unrestricted tumor recognition and a strong safety profile position them as an ideal chassis for universal, next-generation immunotherapies, with demonstrated cytotoxicity against glioblastoma cell lines (153, 154). This section consolidates current knowledge on the therapeutic application of γδ T cells, focusing on their sourcing for clinical use, the mechanistic underpinnings of their CAR-engineered counterparts, strategies to navigate bioengineering limitations, and the current state of clinical translation.

#### 4.3.1 Cell source and *ex vivo* expansion

A foundational challenge for any ACT modality is the generation of a sufficient number of functional effector cells for clinical dosing. The principal source for γδ T cell manufacturing is PBMCs, from which these lymphocytes can be isolated for either autologous or allogeneic applications. Human γδ T cells comprise two major subsets with distinct anatomical distributions and biological roles. The Vγ9Vδ2 subset is the predominant population in peripheral circulation and is uniquely responsive to phosphoantigens (pAgs), which are metabolic intermediates frequently upregulated in transformed cells (155). In contrast, Vδ1-expressing T cells are largely tissue-resident, where they perform immunosurveillance within epithelial and mucosal tissues.

The low physiological frequency of γδ T cells necessitates robust ex vivo expansion methodologies. For the Vγ9Vδ2 subset, proliferation is commonly induced using aminobisphosphonates, such as zoledronate, which inhibit the mevalonate pathway in accessory cells. This blockade leads to the accumulation of isopentenyl pyrophosphate (IPP), a potent endogenous pAg that, in the presence of cytokines like IL-2 and IL-15, triggers selective and large-scale expansion of Vγ9Vδ2 T cells (156). The expansion of Vδ1 T cells and polyclonal γδ T cell populations is less straightforward, often relying on stimulation with TCR agonists like anti-CD3 antibodies, cytokine cocktails, or artificial antigen-presenting cells. Umbilical cord blood has also been identified as a valuable alternative source, providing a naive and potentially more plastic pool of progenitors for therapeutic development. The selection of a starting cell source and an expansion protocol is a critical determinant of the final therapeutic product’s composition, functional attributes, and ultimate clinical potential.

#### 4.3.2 The therapeutic mechanism of CAR-γδ T cells

The therapeutic rationale for CAR-γδ T cells is predicated on a powerful principle of dual recognition, which integrates the bespoke specificity of the engineered CAR with the cell’s intrinsic anti-tumor capabilities. This dual-pronged attack mechanism offers a significant advantage over conventional CAR-αβ T cells, particularly in the context of antigenically heterogeneous solid tumors and the challenge of immune escape.

The primary killing mechanism is directed by the CAR construct, which enables the γδ T cell to recognize a specific TAA in an MHC-independent fashion. This engagement initiates a potent activation cascade, resulting in targeted cytolysis through the release of cytotoxic granules and the secretion of pro-inflammatory cytokines. However, the therapeutic activity of CAR-γδ T cells extends beyond this engineered interaction. γδ T cells are naturally equipped with a diverse array of germline-encoded receptors that allow them to sense and eliminate stressed or malignant cells. The native γδ TCR can recognize stress-induced ligands, including butyrophilin family members (e.g., BTN3A1) by Vγ9Vδ2 T cells. Crucially, γδ T cells also express a suite of receptors typically associated with NK cells, most notably NKG2D (157). This receptor recognizes a family of stress ligands, including MICA/B and ULBPs, that are frequently overexpressed on malignant cells but are largely absent from healthy tissues.

This innate recognition capacity constitutes a vital secondary, and complementary, mechanism of action. If a tumor cell downregulates the target TAA to evade CAR-mediated recognition, its metabolically stressed state often leads to the upregulation of NKG2D ligands, rendering it susceptible to elimination through the γδ T cell’s intrinsic machinery. This dual-targeting paradigm provides a built-in resistance to antigen escape, a major mechanism of relapse following treatment with single-target immunotherapies.

#### 4.3.3 Strategies to overcome limitations in CAR-γδ T cells bioengineering

Realizing the full clinical potential of CAR-γδ T cells requires innovative bioengineering solutions to address limitations related to gene delivery, functional persistence, and therapeutic potency.

Genetic Modification: The introduction of CAR constructs into γδ T cells has historically relied on viral vectors. Lentiviral vectors are often favored due to their ability to transduce non-dividing cells and a more favorable safety profile compared to gamma retroviruses (158). However, to enhance safety and simplify manufacturing, non-viral gene delivery platforms are gaining traction. The Sleeping Beauty transposon system, for example, allows for stable gene integration via electroporation, circumventing the complexities and potential risks of viral vectors. For applications where transient CAR expression is desired to mitigate potential on-target, off-tumor toxicities, mRNA electroporation offers a compelling alternative, enabling potent but time-limited anti-tumor activity.

Enhancing Potency and Persistence: The long-term efficacy of CAR-T therapy is critically dependent on the persistence and sustained function of the engineered cells *in vivo*. The molecular design of the CAR construct itself is a key variable. The choice of intracellular co-stimulatory domains, such as CD28 or 4-1BB, profoundly influences the cell’s metabolic programming, differentiation state, and capacity for memory formation. Additional strategies to bolster γδ T cell function include the co-expression of supportive cytokines like IL-15, which promotes T cell survival and memory development. A particularly insightful strategy involves “arming” CAR-γδ T cells to overcome the immunosuppressive tumor microenvironment. This can be achieved by engineering cells to secrete immune-stimulatory cytokines or by using gene editing to knock out inhibitory receptors, thereby rendering them more resilient in the hostile tumor milieu.

## 5 Clinical trials of cell therapy

NK cells exhibit inherent therapeutic advantages, including MHC-independent target recognition, tumor tissue infiltration capacity, and robust cytolytic activity, while minimizing risks of severe adverse events such as CRS, GvHD, and ICANS. These attributes position CAR-NK cells as a promising modality for addressing solid malignancies. To date, clinical investigations have predominantly focused on CAR-NK products derived from the NK92 cell line, PB-NK, or UCB-K. Current trials registered on ClinicalTrials.gov (summarized in **Table 1**) increasingly target antigens implicated in solid tumors, including NKG2D ligands, mesothelin (MSLN), HER2 and MUC1.

**Table 1:** Clinical trials of CAR-NK cell therapy in solid tumors.

National Clinical Trial (NCT) number	Title	Status	Conditions	CAR-NK product	Targeted antigen	Modification to overcome the limitations	Phase	Start date	Sample size	Primary endpoints
NCT06454890	Clinical Study of Trop2 CAR-NK in the Treatment of Relapsed/Refractory Non-Small Cell Lung Cancer (OC)	Not yet recruiting	Relapsed /Refactory non-small cell lung cancer	Anti-Trop2 U-CAR-NK cells	TROP2	Targeting Trop2 which are positive in tumor tissues	Phase 1 Phase 2	1-Aug-2024	50	Safety (up to 1 year, CTCAE V5.0: Trop2 CAR-NK - related AEs); ORR (up to 1 year, RECIST 1.1: CR/PR pre - progression/treatment)
NCT05922930	Study of TROP2 CAR Engineered IL15-transduced Cord Blood-derived NK Cells Delivered Intraperitoneally for the Management of Platinum Resistant Ovarian Cancer, Mesonephric-like Adenocarcinoma, and Pancreatic Cancer	Recruiting	High grade serous ovarian cancer	Anti-Trop2 U-CAR-NK cells	TROP2	Targeting Trop2 which are positive in tumor tissues	Phase 1 Phase 2	11-Oct-2023	51	Adverse event incidence (up to 1 year, NCI CTCAE v5.0);Safety, optimal dose & MTD/RP2D of intraperitoneal TROP2-CAR/IL15-CB-NK (up to 1 year)
NCT06066424	Phase 1 Dose Escalation and Expansion Study of TROP2 CAR Engineered IL15-transduced Cord Blood-derived NK Cells in Patients With Advanced Solid Tumors (TROPIKANA)	Recruiting	Advanced forms of solid tumors	Anti-Trop2 U-CAR-NK cells	TROP2	Targeting Trop2 which are positive in tumor tissues	Phase 1	24-Oct-2023	54	Adverse event incidence (up to 1 year, NCI CTCAE v5.0);Safety, tolerability, OCD, MTD & RP2D of TROP2-CAR-NK (up to 1 year, for high TROP2-expressing solid tumors
NCT05776355	NKG2D CAR-NK Cell Therapy for Patients With Platinum-Resistant Recurrent Ovarian Cancer	Recruiting	Platinum-resistant, relapsed epithelial ovarian cancer	CAR-NK cells targeting NKG2D ligands	NKG2DL	Allogeneic CAR-NK cells	Not Applicable	1-Mar-2023	18	DLT (within 28 days);MTD (within 28 days) of NKG2D CAR - NK for platinum - resistant recurrent ovarian cancer
NCT06478459	Endoscopic Ultrasound (EUS) Intratumoral Injection of CAR-NK Cells in the Treatment of Advanced Pancreatic Cancer	Recruiting	Advanced pancreatic cancer	CAR-NK cells targeting NKG2D ligands	NKG2DL	Allogeneic CAR-NK cells	Early Phase 1	9-Jun-2024	20	MTD (within 28 days after NKG2D CAR-NK treatment); Incidence of dose-limiting toxicity (up to 2 years)
NCT05213195	NKG2D CAR-NK Cell Therapy in Patients With Refractory Metastatic Colorectal Cancer	Recruiting	Refractory metastatic colorectal cancer	CAR-NK cells targeting NKG2D ligands	NKG2DL	Allogeneic CAR-NK cells	Phase 1	10-Dec-2021	38	DLT (within 28 days); MTD (within 28 days)
NCT03415100	Pilot Study of NKG2D-Ligand Targeted CAR-NK Cells in Patients With Metastatic Solid Tumors	Unknown status	Metastatic solid tumors	CAR-NK cells targeting NKG2D ligands	NKG2DL	Allogeneic CAR-NK cells	Phase 1	2-Jan-2018	30	Number of Adverse Events (from day 0 - month 4)
NCT05248048	NKG2D CAR-T Cells to Treat Patients With Previously Treated Liver Metastatic Colorectal Cancer	Unknown status	Previously treated liver metastatic colorectal cancer	CAR-NK cells targeting NKG2D ligands	NKG2DL	Allogeneic CAR-NK cells	Early Phase 1	13-Sep-2021	9	DLT (within 28 days); MTD (within 28 days)
NCT06503497	A Trail of Second-line Chemotherapy Sequential NKG2D CAR-NK Cell Therapy for Pancreatic Cancer	Recruiting	Pancreatic cancer	CAR-NK cells targeting NKG2D ligands	NKG2DL	Allogeneic CAR-NK cells	Early Phase 1	9-Jul-2024	30	MTD (within 28 days)
NCT05528341	NKG2D-CAR-NK92 Cells Immunotherapy for Solid Tumors	Recruiting	Relapsed/refractory solid tumors	NKG2D CAR-NK92 cells	NKG2DL	Off-the-shelf NK92 cell line-based CAR-NK	Phase 1	26-Jan-2023	20	Safety evaluation (within 3 months); Objective Response Rate (up to 1 year)
NCT05507593	Study of DLL3-CAR-NK Cells in the Treatment of Extensive Stage Small Cell Lung Cancer	Unknown status	Extensive stage small cell lung cancer	CAR-NK cells targeting DLL3	DLL3	Targeting DLL3 which are expressed on the surface of small cell lung cancer tumor cells.	Phase 1	1-Sep-2022	18	DLT (within 28 days); MTD (within 28 days)
NCT05410717	CLDN6-CAR-NK cell therapy for advanced solid tumors	Recruiting	Advanced ovarian cancer or other cancers with expression of claudin6	Claudin6 targeting CAR-NK cells	Claudin6	Engineered to express IL7/CCL19 and/or scfv against PD1/CTLA4/Lag3	Phase 1	1-Jun-2022	200	Safety evaluated by Common Terminology Criteria for Adverse Events (CTCAE) V5.0 (up to 52 weeks after CAR - NK cells infusion)
NCT03941457	Clinical Research of ROBO1 Specific BiCAR-NK Cells on Patients With Pancreatic Cancer	Unknown status	Pancreatic cancer	ROBO1 CAR-NK cells	ROBO1 (Roundabout homolog 1)	Off-the-shelf NK92 cell line-based CAR-NK	Phase 1 Phase 2	1-May-2019	9	Occurrence of treatment-related adverse events as assessed by CTCAE v4.03 (within 1 year)
NCT03940820	Clinical Research of ROBO1 Specific CAR-NK Cells on Patients With Solid Tumors	Unknown status	Solid tumor-pancreatic cancer	ROBO1 CAR-NK cells	ROBO1 (Roundabout homolog 1)	Off-the-shelf NK92 cell line-based CAR-NK	Phase 1 Phase 2	1-May-2019	20	Occurrence of treatment - related adverse events as assessed by CTCAE v4.03 (within 1 year)
NCT05194709	Study of Anti-5T4 CAR-NK Cell Therapy in Advanced Solid Tumors	Unknown status	Advanced solid tumors	Anti-5T4 CAR-NK cells	Oncofetal trophoblast glyprotein (5T4)	Targeting 5T4 (oncofetal antigen) which allow survival of tumor in its host	Early Phase 1	30-Dec-2021	40	Number of Adverse Events (AEs) (From day 1 to day 90 after the last dose)
NCT05137275	Study of Anti-5T4 CAR-raNK Cell Therapy in Locally Advanced or Metastatic Solid Tumors	Unknown status	Locally advanced or metastatic solid tumors	Anti-5T4 CAR-NK (allogeneic NK) cells	Oncofetal trophoblast glyprotein (5T4)	Allogeneic CAR-NK cells	Early Phase 1	24-Nov-2021	56	Incidence of dose limiting toxicity DLTs); Number of Adverse Events (AEs); Objective response rate (ORR); Disease control rate (DCR); Duration of remission (DOR);Progression-free survival (PFS);Overall survival (OS)
NCT06464965	Clinical Study of Cord Blood-Derived CAR-NK Cells in Gastric Cancer and Pancreatic Cancer	Recruiting	Advanced gastric cancer and advanced pancreatic cancer	CB CAR-NK182	Claudin18.2	Targeting Claudin18.2 which are expressed on the surface of cancer tumor cells.	Phase 1	19-Jul-2024	30	DLT (within 28 days); MTD (within 28 days)
NCT06652243	Clinical Study of SN301A Injection in the Treatment of Hepatocellular Carcinoma (SN301A)	Recruiting	Advanced hepatocellular carcinoma	CAR-NK cells targeting GPC3	GPC3	Targeting GPC3 which are positive in tumor tissues	Early Phase 1	18-Nov-2024	12	DLT (28 days post SN301A infusion); Incidence and severity of adverse events and serious adverse events (through study completion, up to 2 years)
NCT04847466	Immunotherapy Combination: Irradiated PD-L1 CAR-NK Cells Plus Pembrolizumab Plus N-803 for Subjects With Recurrent/Metastatic Gastric or Head and Neck Cancer	Recruiting	Gastric or head and neck cancer	PD-L1 CAR-NK cells	PD-L1	Irradiated PD-L1 CAR-NK cells, combined with pembrolizumab and N-803	Phase 2	14-Dec-2021	55	Clinical response rate (CR + PR) every 6 weeks
NCT03692637	Study of Anti-Mesothelin CAR-NK Cells in Epithelial Ovarian Cancer	Unknown status	Epithelial ovarian cancer	Anti-Mesothelin CAR-NK Cells	MSLN	PBMC-derived anti-mesothelin CAR-NK	Early Phase 1	1-Mar-2019	30	Occurrence of treatment-related adverse events as assessed by CTCAE v4.0 (From day 3 to year 2 after injection)
NCT02839954	CAR-pNK Cell Immunotherapy in MUC1 Positive Relapsed or Refractory Solid Tumor	Unknown status	MUC1 positive relapsed or refractory solid tumor	Anti-MUC1 CAR-pNK cells	MUC1	Targeting MUC1 on epithelial surfaces of enhanced tumor infiltration	Phase 1 Phase 2	1-Jul-2016	10	Adverse events attributed to the administration of the anti-MUC1 CAR-pNK cells (within 2 years)
NCT03692663	Study of Anti-PSMA CAR-NK Cell (TABP EIC) in Metastatic Castration-Resistant Prostate Cancer	Unknown status	Metastatic castration-resistant prostate cancer	Anti-PSMA CAR-NK cells	PSMA	Targeting PSMA which are positive in tumor tissues	Early Phase 1	1-Dec-2018	9	Occurrence of treatment - related adverse events as assessed by CTCAE v5.0 (From baseline to 1 year post infusion)

To date, early-phase clinical investigations of CAR-Ms remain limited, with only a small number of registered trials documented on ClinicalTrials.gov (summarized in **Table 2**). The pioneering Phase I trial (NCT04660929), designated CT-0508, evaluates the safety and feasibility of CAR-M therapy in HER2-overexpressing solid tumors. Developed by CARISMA Therapeutics, this candidate utilizes a chimeric adenoviral vector (Ad5f35) for ex vivo genetic modification of autologous PBMC-derived macrophages, enabling HER2-targeted antitumor activity. CT-0508 represents the first-in-human application of CAR-Ms, with preliminary objectives focused on assessing dose-limiting toxicities, pharmacokinetics, and biomarker correlates of response. The trial design incorporates dose escalation followed by expansion cohorts, with planned combinatorial arms exploring synergy with checkpoint inhibitors. Preclinical data underpinning this trial demonstrated that Ad5f35-engineered CAR-Ms not only directly phagocytose HER2+ tumor cells but also remodel immunosuppressive microenvironments via pro-inflammatory cytokine secretion, thereby enhanad5cing T cell infiltration.

**Table 2:** Clinical trials of CAR-M cell therapy in solid tumors.

National Clinical Trial (NCT) number	Title	Status	Conditions	CAR-M product	Targeted antigen	Modification to overcome the limitations	Phase	Start date	Sample size	Primary endpoints
NCT06224738	Human HER2-targeted Macrophages Therapy for HER2-positive Advanced Gastric Cancer With Peritoneal Metastases	Not yet recruiting	Advanced HER2+ gastric cancer	Human HER2-targeted CAR-M cells	HER2	Autologous macrophages express CAR-Molecules containing single-chain antibodies which specifically bind to human HER2 antigens	Early Phase 1	1-Mar-2024	9	Adverse effects (evaluated within 28 days of administration, with a total of 30 recording points)
NCT04660929	CAR-macrophages for the Treatment of HER2 Overexpressing Solid Tumors	Active, not recruiting	HER2 overexpressing solid tumors	Human HER2-targeted CAR-M cells	HER2	Autologous Macrophages Engineered to Contain an Anti-HER2 CAR	Phase 1	2-Feb-2021	48	Adverse events (including CRS) frequency/severity; Products passing release criteria percentage; Adverse events (including CRS) frequency/severity in CT-0508 + pembrolizumab substudy
NCT05007379	Cohort Study to Determine the Antitumor Activity of New CAR-macrophages in Breast Cancer Patients’ Derived Organoids (CARMA)	Unknown status	Breast cancer patients’ derived organoids	New CAR-M cells	HER2	Cell-based immune therapy using modified macrophages	/	1-Sep-2021	100	CAR-macrophages’ antitumor activity against HER2-neg/low/pos breast cancer organoids (within 24 months); CAR-macrophages’ antitumor activity vs non-modified macrophages (within 24 months)
NCT06562647	SY001 Targets Mesothelin in a Single-arm, Dose-increasing Setting in Subjects With Advanced Solid Tumors	Recruiting	Advanced solid tumors	CAR-pMAC cells	MSLN	PBMC-derived anti-mesothelin CAR-M	Not Applicable	15-Aug-2024	2	MTD (up to 28 days after SY001 infusion)

The clinical translation of γδ T cell therapy has progressed from early-phase trials utilizing unmodified cells to contemporary studies evaluating advanced, genetically engineered products. Initial clinical studies focused on either activating endogenous Vγ9Vδ2 T cells *in vivo* with agents like zoledronate and low-dose IL-2, or the adoptive transfer of ex vivo-expanded autologous γδ T cells (159). These foundational trials, conducted in patients with diverse solid and hematological cancers, consistently demonstrated an exceptional safety profile. The therapies were well-tolerated, with a notable absence of GvHD, even in the allogeneic setting. While these studies established safety and feasibility, objective response rates were modest, with disease stabilization being the most common outcome (160).

The crucial safety data from these early efforts provided the impetus for developing the next generation of γδ T cell therapies. A pivotal evolution has been the shift towards allogeneic, “off-the-shelf” products derived from healthy donors, a strategy that leverages the innate safety of γδ T cells to create a standardized, readily available therapeutic. Current clinical trials are increasingly focused on CAR-γδ T cells. Early-phase studies are now underway to evaluate the safety and preliminary efficacy of these constructs in both blood cancers and solid tumors, targeting antigens such as CD19, CD20, and NKG2D ligands. The results from these trials will be instrumental in validating the clinical potential of engineered γδ T cells and will inform the design of future, more sophisticated therapeutic strategies aimed at fully harnessing their potent anti-cancer capabilities. To date, only a few clinical studies evaluating CAR- γδ T cell products have been registered on ClinicalTrials.gov, as summarized in **Table 3**.

**Table 3:** Clinical trials of CAR- γδ T cell therapy in solid tumors.

National Clinical Trial (NCT) number	Title	Status	Conditions	CAR-γδ T product	Targeted antigen	Modification to overcome the limitations	Phase	Start date	Sample size	Primary endpoints
NCT06150885	A Safety and Efficacy Study of Allogeneic CAR Gamma-Delta T Cells in Subjects with Relapsed/​Refractory Solid Tumors (CAR001)	Recruiting	Relapsed/Refractory solid tumors	HLA-G-CAR.BiTE allogeneic γδ T cells	ER, PR, HER-2	Using allogeneic chimeric antigen receptor (CAR) Gamma-Delta T cells	Phase 1 Phase 2	1-Sep-2024	60	MTD of CAR001 (4 weeks after last dosing of CAR001);Objective Response Rate (ORR)(from visit 1 to 24 - months of safety and efficacy follow - up period)
NCT06372236	UTAA06 Injection for Treatment of Advanced Malignant Solid Tumors	Recruiting	Advanced malignant solid tumors	CAR+ γδ T cells	B7-H3	Targeting B7-H3 which is overexpressed on a variety of solid tumors.	Phase 1	1-Dec-2023	10	MTD (about 2 years);Overall response rate (ORR) at 3 months (about 2 years, based primarily on solid tumor response criteria RECISTv1.1) Number of participants with treatment - related adverse events as assessed by CTCAE V5.0 (about 2 years)
NCT06193486	Autologous Gamma Delta T Cells to Target Prostate Stem Cell Antigen in mCRPC	Recruiting	MCR prostate cancer	Autologousγδ T Cells Genetically Engineered with a Chimeric Receptor	PSCA	PSCA-specific CAR-transduced γδ T cells	Phase 1	26-Feb-2024	30	Maximum Tolerated Dose (MTD) of MSGV1-PSCA-8T28Z (Up to 30 days post transplant)
NCT05302037	Allogeneic NKG2DL-targeting CAR γδ T Cells (CTM-N2D) in Advanced Cancers (ANGELICA)	Recruiting	Solid and hematological tumors	Allogeneic NKG2DL-targeting CAR-grafted γδ T Cells	NKG2DL	NKG2D ligand-specific CAR-transduced Vγ9Vδ2 T cells (CTM-N2D)	Phase 1	19-Nov-2024	12	CTM-N2D dose regimen/schedule (≤1 DLT in 6 patients at same dose level, within 8 weeks post study treatment start);Number of treatment-related AEs (24 months post study treatment start)

## 6 Application of CAR-NK and CAR-M in gynecologic tumors

The therapeutic potential of CAR-NK, CAR-M, and CAR-γδ T cells in solid tumors is driven by their divergent mechanisms. CAR-NK cells exhibit a “dual-modal killing” mechanism. Firstly, through their chimeric antigen receptor, they release perforin and granzymes to mediate direct cytotoxicity and secrete IFN-γ to modulate TME. Secondly, they retain innate ADCC via their CD16 receptor, which enables the elimination of tumor cells that downregulate the CAR-targeted antigen, thereby counteracting tumor heterogeneity (90). CAR-M cells are primarily characterized by their potent phagocytic capacity. A central feature of their function is the ability to remodel the immunosuppressive TME. They secrete pro-inflammatory cytokines including TNF-α and IL-6, recruit cytotoxic T cells, enhance antigen presentation, and can polarize macrophages from the M2 to the M1 phenotype in the TME (161). CAR-γδ T cells combine features of both innate and adaptive immunity. They kill target cells through perforin and granzyme release as well as death receptor-mediated apoptosis such as via the Fas/FasL pathway. A hallmark of these cells is their MHC-independent recognition of stress-induced ligands through receptors like NKG2D, which provides a critical advantage against tumors that evade MHC-dependent recognition (162).

CAR therapies for the above cells are already in the clinical stage, and this paragraph mainly lists CAR-NK and CAR-M in gynecological tumors (e.g., OC). In preclinical studies of OC, CAR-NK targeting HAL-G, CD44, MSLN and αFR has demonstrated superior antitumor effects (163–167). Zhu et al. generated ROBO1-targeted CAR NK cells from PBMCs of OC patients. Efficacy was evaluated using xCELLigence RTCA, CCK-8 and Live/Dead fluorescence assays. Compared with primary NK cells without ROBO1-CAR modification (168), ROBO1-NK cells exhibited higher efficiency in eradicating primary ovarian cancer cells and lysing ovarian tumor organoids. Raftery et al. developed a next-generation CAR targeting CD44v6 that incorporates IL-15 superagonist and checkpoint inhibitor molecules. It could show that CD44v6 CAR-NK cells demonstrated effective cytotoxicity against triple negative breast carcinoma (TNBC) in 3D spheroid models (169). Kutle et al. engineered NK cells with a third-generation CAR targeting MSLN, a tumor-associated antigen overexpressed in primary human cervical carcinomas and established cell lines, as evidenced by their study and prior research. The CAR construct, delivered via self-inactivating (SIN) alpha retroviral vectors, incorporated tandem co-stimulatory domains (CD28 and 4-1BB) to enhance NK cell activation and persistence. In functional assays, anti-MSLN CAR-NK-92 cells exhibited potent cytotoxicity against cervical cancer models, with efficacy validated in both 2D monolayers and 3D spheroid cultures. This antitumor activity correlated with elevated degranulation markers (e.g., CD107a) in CAR-NK-92 cells upon MSLN+ target engagement. To confirm antigen-specific targeting, the authors employed CRISPR-Cas9 gene editing to generate MSLN-knockout cervical cancer cells. Co-culture experiments with these isogenic pairs demonstrated that cytotoxic activity of both CAR-NK-92 cells and primary CAR-NK cells (derived from healthy donors) was strictly dependent on MSLN expression. Furthermore, combinatorial treatment with anti-MSLN CAR-NK-92 cells and conventional chemotherapeutic agents synergistically enhanced tumor cell elimination compared to monotherapy regimens, suggesting potential for integrated therapeutic strategies (170). Poorebrahim et al. engineered a dual-receptor system in NK-92 cells by integrating a clinically validated TCR targeting human papillomavirus 16 (HPV16) E7 oncoprotein with a novel CAR directed against trophoblast cell surface antigen 2 (TROP2). The CAR construct incorporated CD28 and 4-1BB co-stimulatory domains but excluded the CD3ζ signaling module, a design choice aimed at modulating activation thresholds. Flow cytometric analysis revealed significant upregulation of activation markers (e.g., CD69, CD25) and cytolytic mediators (e.g., granzyme B) in NK-92 cells co-expressing CD3, CD8, E7-TCR, and TROP2-CAR following exposure to HPV16+ cervical cancer cells. Notably, dual-receptor NK-92 cells exhibited superior antigen-specific activation and tumoricidal activity compared to counterparts expressing E7-TCR alone. This enhancement was attributed to synergistic signaling between the E7-TCR and TROP2-CAR, wherein the co-stimulatory domains (CD28/4-1BB) amplified intracellular activation cascades. Mechanistically, TROP2-CAR engagement augmented TCR-mediated cytotoxicity without inducing exhaustive phenotypes, suggesting a balanced signaling interplay. This combinatorial strategy holds translational promise for HPV16-associated malignancies, potentially overcoming limitations of single-receptor adoptive therapies. By coupling viral antigen specificity (via TCR) with tumor-associated antigen targeting (via CAR), the approach may broaden targetable epitopes while mitigating antigen escape. Current clinical investigations are evaluating such engineered NK cells in HPV16+ cancers, with implications for improving therapeutic efficacy and durability (171). Klapdor et al. developed a CAR-engineered immunotherapy targeting CD133, a well-established CSC marker, to address therapeutic challenges in OC. The researchers constructed a third-generation anti-CD133 CAR incorporating CD28 and 4-1BB co-stimulatory domains, which was encoded into a lentiviral vector for stable genetic modification. Clinically applicable NK92 cells—a standardized natural killer cell line—were transduced with this construct to generate CD133-specific CAR-NK cells. Functional validation assays demonstrated selective cytotoxicity of engineered CAR-NK92 cells against CD133-positive OC cell lines *in vitro*. Notably, this activity extended to primary OC cells isolated from sequential ascites samples of patients with advanced disease, underscoring the therapeutic relevance of targeting CD133-expressing tumor populations. The observed antitumor efficacy correlated with CSC depletion, suggesting potential to mitigate relapse driven by chemotherapy-resistant stem-like cells (172).

The HER2 gene is closely associated with tumorigenesis, particularly in breast cancer, and is amplified across multiple solid malignancies. In OC, HER2 overexpression correlates with aggressive phenotypes, including recurrence and metastasis, while maintaining minimal expression in healthy tissues (173). Leveraging this therapeutic window, Chen et al. engineered CAR-Ms dually targeting HER2 and CD47, a “don’t eat me” signal, to enhance tumor-specific phagocytosis. *In vitro* assays demonstrated that HER2/CD47-targeted CAR-Ms selectively engulfed OC cells and activated CD8+ cytotoxic T lymphocytes (CTLs) via pro-inflammatory cytokine secretion, thereby fostering adaptive antitumor immunity. In humanized mouse models, CAR-M administration induced tumor regression, concomitant with enhanced CD8+ T cell activation and polarization of TAMs toward pro-inflammatory phenotypes (29).

Immune cell therapy has emerged as a transformative strategy to bolster immune responses and counteract tumor-induced immunosuppression. This approach can be utilized either independently or in combination with established conventional therapies, such as surgery, radiotherapy, chemotherapy, and other immunotherapeutic modalities, for treatment in gynecological cancers. By effectively reducing tumor recurrence and metastasis rates while mitigating adverse drug reactions, immune cell therapy offers a promising avenue to enhance patient survival outcomes. Intriguingly, this innovative therapeutic paradigm holds immense potential for extending to women with gynecologic malignancies for improving therapeutic efficacy in future clinical settings.

## 7 Outlook

While CAR-T cell therapy has demonstrated remarkable clinical efficacy in hematological malignancies, persistent challenges hinder its broader application. Critical limitations include CRS, ICANS, antigen escape mechanisms, poor adaptation to immunosuppressive TMEs, high relapse rates, and prohibitive manufacturing costs. These shortcomings have spurred exploration of alternative immune effector platforms, notably CAR-NK cells and CAR-Ms, which exhibit distinct therapeutic advantages.

CAR-NK cells offer intrinsic benefits such as non-MHC-restricted cytotoxicity, potential for “off-the-shelf” allogeneic use, reduced risk of CRS/ICANS, and lower production costs compared to patient-specific CAR-T products. Furthermore, their limited *in vivo* persistence may mitigate long-term toxicity risks. CAR-Ms, conversely, demonstrate unique tropic capabilities to penetrate immunosuppressive TMEs and remodel stromal barriers, positioning them as promising agents for solid tumor eradication. These attributes collectively advocate for CAR-NK and CAR-M therapies as viable alternatives to circumvent CAR-T limitations while maintaining robust antitumor activity.

To optimize CAR-based immunotherapies, emerging strategies prioritize:

Target Antigen Innovation: High-throughput screening for tumor-specific surface markers with minimal on/off-tumor cross-reactivity;
TME reprogramming: Co-expression of immunomodulatory payloads (e.g., cytokines, checkpoint inhibitors) to counteract immunosuppression (174, 175);
Combinatorial regimens: Synergistic pairing with radiotherapy, chemotherapy, or targeted therapies to enhance CAR cell infiltration and durability;
Architectural refinement: Engineering next-generation CAR constructs (e.g., logic-gated receptors, hypoxia-responsive switches) tailored for solid tumor biology. New approaches include hypoxia-sensitive CARs activated under tumor conditions, logic-gated CARs requiring dual-antigen recognition, and CAR constructs with built-in safety switches for toxicity control (176). Together, these designs highlight innovative engineering tactics to enhance the specificity and safety of CAR therapies in solid tumors (177).

Preclinical studies and early-phase trials have validated the feasibility of these approaches, underscoring the necessity for continued translational investment. Key barriers to translating CAR-immunocellular therapies from preclinical to clinical success include: inefficient tumor infiltration, immunosuppressive tumor microenvironments, antigen heterogeneity/escape, on-target/off-target toxicity, and limited survival of CAR-NK/CAR-M/CAR-γδ T cells in solid tumors. In this regard, combining CAR engineering with the intrinsic ability of NK and γδ T cells to recognize stress-induced ligands (e.g., MICA/B, ULBPs, BTN2A1/BTN3A1) offers a dual-targeting strategy that may reduce antigen escape and enhance therapeutic safety and efficacy in solid tumors (178). For successful clinical translation, future work must establish standardized protocols for generating clinical-grade CAR-NK/M/γδ T products from patient-derived monocytes.

The development of allogeneic “off-the-shelf” CAR therapies is complex due to their dual classification as both gene and cell products. Early engagement with regulators is essential to establish appropriate potency assays, quality controls, and long-term safety monitoring. Economically, the high cost of current autologous CAR-T therapies underscores the need for cost-effectiveness analyses of allogeneic alternatives. Demonstrating superior value, not just efficacy, will be vital for reimbursement and patient access.

Future research must overcome these fundamental barriers to fully unlock the clinical potential of emerging platforms such as CAR-NK, CAR-M, and CAR-γδ T cells.

## Glossary

ACT: Adoptive cell therapy
CAR: chimeric antigen receptor
TME: tumor microenvironment
CRS: cytokine release syndrome
CAR-NK: CAR natural killer
HLA: human leukocyte antigen
CAR-M: CAR-macrophage
TAMs: tumor-associated macrophages
CAR-γδ T: CAR-Gamma-Delta T
MHC: major histocompatibility complex
MHC-I: major histocompatibility complex I
PBMCs: peripheral blood mononuclear cells
ICANS: immune effector cell-associated neurotoxicity syndrome
OC: ovarian cancer
HCC: hepatocellular carcinoma
HNSCC: head and neck squamous cell carcinoma
HER2: human epidermal growth factor receptor 2
IFN-γ: interferon-γ
PDAC: pancreatic ductal adenocarcinoma
IL: interleukin
TNF-α: tumor necrosis factor-α
PRRs: pattern-recognition receptors
DCs: dendritic cells
MAIT: mucosa-associated invariant T
TCR: T-cell receptor
FcRs: Fc receptors
ADCC: antibody-dependent cellular cytotoxicity
ADCP: antibody-dependent cellular phagocytosis
scFv: single-chain variable fragment
VH: variable heavy
VL: variable light
ITAMS: immunoreceptor tyrosine-based activation motifs
DAP10: DNAX activation protein of 10 kDa
DAP12: DNAX activation protein of 12 kDa
NKG2D: natural killer group 2 member D
EGFR: epidermal growth factor receptor
PD-L1: programmed cell death ligand 1
SLAM: signaling lymphocytic activation molecule
SAP: SLAM-associated protein
iPSC: induced pluripotent stem cell
EBV: Epstein-Barr virus
LCLs: lymphoblastoid cell lines
CXCR4: CXC chemokine receptor 4
TIR: Toll/IL-1R
iMACs: induced pluripotent stem cell-derived macrophages
TLR4: Toll-like receptor 4
MHC-II: MHC class II
MMPs: matrix metalloproteinases
ECM: extracellular matrix
ROS: reactive oxygen species
CCL19: C-C motif chemokine ligand 19
CCR7: C-C motif chemokine receptor
HPCs: hematopoietic progenitor cells
LMPPs: lymphoid-primed multipotent progenitors
CLPs: common lymphoid progenitors
NKPs: NK cell precursors
mNKs: mature NK cells
ELPs: Early lymphoid precursors
PB: peripheral blood
UCB: umbilical cord blood
NKp44: Natural Killer Cell p44-Activating Receptor
GMP: Good Manufacturing Practice
PB-NK: PBMC-sourced NK cells
GvHD: graft-versus-host disease
UCB-NK: umbilical cord blood-derived NK cells
KIR: killer immunoglobulin-like receptors
NCRs: Natural cell toxicity receptors
DNAM-1: DNAX accessory molecule-1
CSCs: cancer stem cells
MICA/B: MHC class I chain-related proteins A/B
NSCLC: Non-small-cell lung cancer
aNK-DLI: activated NK cell infusions
SCF: stem cell factor
Flt3L: FMS-like tyrosine kinase 3 ligand
TPO: thrombopoietin
G-CSF: granulocyte colony-stimulating factor
GM-CSF: granulocyte-macrophage CSF
SB: Sleeping Beauty
ITRs: inverted terminal repeats
HSPCs: hematopoietic stem/progenitor cells
gRNA: guide RNA
*mbIL-15*: membrane-bound interleukin-15
DKI: double knock-in
iNK: iPSC-NK
TGF-β: transforming growth factor
Th1: Type 1 T helper cell
Th2: Type 2 T helper cell
MPEI: mannose-conjugated polyethyleneimine
LNPs: Lipid Nanoparticles
IPP: isopentenyl pyrophosphate
TAAs: tumor-associated antigen
MSLN: mesothelin
MUC1: mucin 1
TNBC: triple negative breast carcinoma
SIN: self-inactivating
HPV16: human papillomavirus 16
TROP2: trophoblast cell surface antigen 2
CTLs: cytotoxic T lymphocytes
